# Docetaxel remodels prostate cancer immune microenvironment and enhances checkpoint inhibitor-based immunotherapy

**DOI:** 10.7150/thno.73152

**Published:** 2022-06-27

**Authors:** Zehua Ma, Weiwei Zhang, Baijun Dong, Zhixiang Xin, Yiyi Ji, Ruopeng Su, Kai Shen, Jiahua Pan, Qi Wang, Wei Xue

**Affiliations:** 1 Department of Urology, Ren Ji Hospital, Shanghai Jiao Tong University School of Medicine, Shanghai 200120, China.; 2 Shanghai Key Laboratory for Tumor Microenvironment and Inflammation, Shanghai Jiao Tong University School of Medicine, Shanghai 200120, China.

**Keywords:** Prostate Cancer, Docetaxel, cGAS/STING, Immunotherapy, Immune microenvironment

## Abstract

**Background:** Prostate cancer is usually considered as immune “cold” tumor with poor immunogenic response and low density of tumor-infiltrating immune cells, highlighting the need to explore clinically actionable strategies to sensitize prostate cancer to immunotherapy. In this study, we investigated whether docetaxel-based chemohormonal therapy induces immunologic changes and potentiates checkpoint blockade immunotherapy in prostate cancer.

**Methods:** We performed transcriptome and histopathology analysis to characterize the changes of prostate cancer immune microenvironment before and after docetaxel-based chemohormonal therapy. Furthermore, we investigated the therapeutic benefits and underlying mechanisms of chemohormonal therapy combined with anti-PD1 blockade using cellular experiments and xenograft prostate cancer models. Finally, we performed a retrospective cohort analysis to evaluate the antitumor efficacy of anti-PD1 blockade alone or in combination with docetaxel-based chemotherapy.

**Results:** Histopathology assessments on patient samples confirmed the enrichment of tumor-infiltrating T cells after chemohormonal therapy. Moreover, we found that docetaxel activated the cGAS/STING pathway in prostate cancer, subsequently induced IFN signaling, resulting in lymphocytes infiltration. In a xenograft mouse model, docetaxel-based chemohormonal therapy prompted the intratumoral infiltration of T cells and upregulated the abundance of PD1 and PD-L1, thereby sensitizing mouse tumors to the anti-PD1 blockade. To determine the clinical significance of these results, we retrospectively analyzed a cohort of 30 metastatic castration-resistant prostate cancer patients and found that docetaxel combined with anti-PD1 blockade resulted in better prostate-specific antigen progression-free survival when compared with anti-PD1 blockade alone.

**Conclusions:** Our study demonstrates that docetaxel activates the antitumoral immune response and facilitates T cell infiltration in a cGAS/STING-dependent manner, providing a combination immunotherapy strategy that would improve the clinical benefits of immunotherapy.

## Introduction

Prostate cancer is the second most prevalent type of cancer and the fifth leading cause of cancer-related death in men worldwide 1. Nowadays androgen-deprived therapies and docetaxel-based chemotherapy are still the standard treatments for advanced prostate cancer patients 2. Nevertheless, given sufficient time, patients will fail treatment and relapse with castration-resistant prostate cancer, among which more than 50% of patients do not respond to docetaxel, highlighting the urgent need for novel therapeutic strategies 3. The past year has witnessed outstanding developments in researches on checkpoint blockade immunotherapy, in particular antibodies that inhibit the PD1 pathway. This strategy proves to be more effective for the so-called “hot” tumor, which harbors a high degree of pre-existing infiltrated lymphocytes 4-6. In contrast, prostate cancer is usually characterized as “cold” tumors with limited infiltrating immune cells and restricted sensibility to checkpoint blockade therapy 7. Hence further exploration is needed to reverse prostate cancer from “cold” to “hot” inflamed tumor, strengthening the benefits from immunotherapy.

Docetaxel is a taxane class of anti-mitotic chemotherapeutic agents. This class of drug preferentially binds to β-tubulin, suppresses microtubule dynamics, disrupts cell division, and therefore effectively induces apoptosis. As an FDA-approved agent, docetaxel has been the standard of care for a variety of cancer types, including advanced prostate cancer 8, 9. Our previous study demonstrated that prostate cancer patients who received docetaxel-based neoadjuvant chemohormonal therapy had better biochemical progression-free survival time after radical prostatectomy, compared with neoadjuvant hormonal therapy group and non-neoadjuvant therapy group 10. The wide use of docetaxel provides an opportunity to understand the impacts of docetaxel on the tumor microenvironment. A phase 3 trial showed that taxane in combination with atezolizumab significantly improved pathological complete response rates in triple-negative breast cancer patients 11. In a non-small cell lung cancer trial, a combination of pembrolizumab plus docetaxel substantially improved overall response rate and progression-free survival. In a mouse model of breast cancer, docetaxel not only inhibited tumor growth but also upregulated CD4^+^ and CD8^+^ T cells proportion via IFN production 12. Another study showed that taxane increased infiltration of CD8^+^ T cells and upregulated PD-L1 expression in ovarian cancer mouse model 13. Above studies prompt us to reconsider the tumor response to docetaxel and explore the possibility of combined treatment with immunotherapy for prostate cancer.

In this study, we aim to investigate how docetaxel-based chemohormonal therapy affects the prostate cancer microenvironment, and whether this effect can activate antitumoral immune response, subsequently benefiting immunotherapy. We obtained prostate cancer samples before and after docetaxel-based chemohormonal therapy and performed whole-transcriptome profiling coupled with histopathology analyses. Docetaxel in combination with hormonal therapy significantly activated the antitumoral immune response and prompted T cell infiltration. Mechanistically, docetaxel treatment upregulates the cGAS/STING pathway in prostate cancer, subsequently activated IFN signaling, resulting in lymphocytes infiltration. We also evaluated the antitumor efficacy of the combined treatment of chemohormonal therapy plus anti-PD1 blockade in a xenograft mouse model. Notably, chemohormonal therapy facilitated T cell infiltration and PD1 and PD-L1 abundance in both patient samples and xenografted tumors. Finally, we performed a retrospective analysis for a cohort of 30 metastatic castration-resistant prostate cancer patients from Renji Hospital and evaluated the antitumor efficacy of anti-PD1 blockade alone or in combination with docetaxel-based chemotherapy. These findings provide a rationale for combination of docetaxel with anti-PD1 blockade to improve cancer therapy.

Our findings show that docetaxel remodels tumor microenvironment by promoting intratumoral infiltration of T cells and upregulating the abundance of PD1 and PD-L1, and show enhanced antitumor activity when combined with anti-PD1 blockade.

## Methods

Immunohistochemistry staining, Immunofluorescence staining, Transcriptome data analysis, QRT-PCR, Detection of DNA, Western blot, Flow cytometry, Plasmid constructs, and Cell growth assay were described in supplementary materials.

### Patients and samples

We collected 86 tumor samples from prostate cancer patients who underwent surgery in Renji hospital. Of the 86 patients, 41 patients received primary radical prostatectomy (Treatment-naive group); 45 patients received docetaxel-based neoadjuvant chemohormonal therapy before radical prostatectomy (Chemohormonal therapy group). The clinicopathologic information for the two groups is summarized in Table S1. 11 of the 45 patients had paired pre- and post-treatment tumor samples in the chemohormonal therapy group, the clinicopathologic information of the 11 patients before and after treatment was summarized in Table S2.

### RNA Sequencing

RNA sequencing was performed on the 11 paired pre- and post-neoadjuvant chemohormonal therapy tumor samples. The clinicopathologic information of the 11 patients before and after treatment, including age, Gleason score, prostate-specific antigen (PSA) level and tumor stage, was summarized in Table S2. Total RNA was extracted using the TRIzol reagent according to the manufacturer's protocol. RNA purity and quantification were evaluated using the NanoDrop 2000 spectrophotometer (Thermo Scientific, USA). RNA integrity was assessed using the Agilent 2100 Bioanalyzer (Agilent Technologies, Santa Clara, CA, USA). Then the libraries were constructed using TruSeq Stranded mRNA LT Sample Prep Kit (Illumina, San Diego, CA, USA) according to the manufacturer's instructions. The transcriptome sequencing and analysis were conducted by OE Biotech Co., Ltd. (Shanghai, China).

The libraries were sequenced on an Illumina HiSeq X Ten platform and 150 bp paired-end reads were generated. Raw reads for each sample were generated. Raw reads of fastq format were firstly processed using Trimmomatic and the low-quality reads were removed to obtain the clean reads. Then clean reads for each sample were retained for subsequent analyses.

### Acquisition of RNA-seq data from Gene Expression Omnibus

The transcriptome data from GSE111177 based on platform GPL16791 was obtained from Gene Expression Omnibus database. GSE111177 contained transcriptome data of 20 paired pre- and post-neoadjuvant hormonal therapy tumor samples 14. We applied the trimmed mean of M values normalization algorithm provided by the edgeR R package to normalize the raw transcriptome data and all gene expression data were log2 transformed 15. The average expression value was accepted for duplicated data. Genes with an average expression value less than 1 were discarded.

### Acquisition of RNA-seq data from The Cancer Genome Atlas Program (TCGA)

RNA-seq data of human prostate cancer samples from TCGA were obtained from TCGA database. The data contained transcriptome data and clinical information of 495 prostate cancer samples. The raw data is normalized using FPKM for batch corrected mRNA gene expression and all gene expression data were log2 transformed. The average expression value was accepted for duplicated data. Genes with an average expression value less than 1 were discarded.

### Cell culture

HEK-293T cells and prostate cancer cell lines including LNCaP, LAPC4, PC3, DU145, and RM1 were purchased from the Cell Bank of Chinese Academy of Sciences (Shanghai, China). Human prostate cancer cells LNCaP and PC3 and mouse prostate cancer cells RM1 were cultured in RPMI medium 1640 (GIBCO; C22400500BT) with 10% fetal bovine serum (GIBCO; A3160802), penicillin (100 U/mL) and streptomycin (100 µg/mL; GIBCO; 15140-122) in a humidified atmosphere of 5% (v/v) CO_2_ in air at 37 °C. HEK-293T cells and human prostate cancer cells DU145 and LAPC4 were cultured in DMEM medium (HyClone; SH30243.01) with 10% fetal bovine serum (GIBCO; A3160802), penicillin (100 U/mL) and streptomycin (100 µg/mL; GIBCO; 15140-122) in a humidified atmosphere of 5% (v/v) CO_2_ in air at 37 °C.

### Mice study

To induce subcutaneous castration-resistant prostate cancer, 5 × 10^4^ RM1 cells were suspended in Opti-MEM/Matrigel (1:1; Corning) and subcutaneously implanted into the right flank of 5 weeks old male C57BL/6J mice. On day 7, the RM1-bearing mice received bilateral castration under anesthesia. On day 10, the subcutaneous tumor volumes of mice reached ~250 mm^2^, mice were randomly assigned into 4 groups, followed by intraperitoneally administration (DMSO, administered intraperitoneally, five times per week for two consecutive weeks, n = 5; anti-PD1 (Bio X cell; RMP1-14), 6 mg/kg per injection, every three days for total four times, n = 5; bicalutamide (Selleck; ICI-176334), 20 mg/kg per injection, five times per week for two consecutive weeks, and docetaxel (Selleck; RP56976), 10 mg/kg per injection, once per week for two consecutive weeks, n = 5; bicalutamide, docetaxel, and anti-PD1, n = 5). Tumor volume was measured every three days. Tumor volumes were calculated as Volume = Length x Width^2^ /2. The mice were sacrificed when the tumor diameter exceeded 2 cm and then the tumor tissues were harvested and divided into several fragments for the future experiments.

To obtain single cells from mice tumors, xenografted tumors were minced with scissors and then enzymatically digested using 0.5 mg/mL collagenase type IV (YEASEN) and 0.01 mg/mL DNase-I (Sangon Biotech) at 37 °C for 30 minutes. Isolated cell suspensions were filtered through a 70 μm cell strainer to obtained single cell suspensions. Single cell suspensions were blocked by Fc block (eBioscience; 14-0161-81) and stained with fixable viability stain 510 (BD; 564406) and following antibodies: CD45 (APC; Biolegend; 103112), CD3 (FITC; BD; 555274), CD8a (BB700; BD; 566409), and PD1 (PE; eBioscience; 12-9985-82). Data were acquired on a LSRFortessa X-20 flow cytometer (BD Biosciences) and further analyzed with FlowJo software.

Formalin-fixed and paraffin-embedded mouse tumor tissue blocks were collected and cut. For immunohistochemistry staining of CD3 (1:200; Abcam; ab16669) and CD8 (1:2000; Abcam; ab217344), 3 µm paraffin-embedded sections were stained. The paraffin-embedded sections or tissue microarray were unmasked in 1 × Tris -EDTA buffer (pH 9.0) for 20 min at 95 °C and then incubated with specific antibodies overnight at 4 °C. Digitalized images were taken using Nikon-80i microscope under 20× objective. For quantification of CD3 and CD8, two independent researchers calculated the average number of membrane-positive cells in five random 20× fields.

All mice experiments were completed in compliance with the requirements of the Shanghai Jiao Tong University School of Medicine, Renji Hospital Ethics Committee.

### Retrospective study

To assess the clinical effect of docetaxel to anti-PD1 immunotherapy, we retrospectively identified 30 metastatic castration-resistant prostate cancer (mCRPC) patients from Renji Hospital, who failed prior novel antiandrogen therapy and did not receive any chemotherapy before. Based on patients' willingness and informed consents, the 30 patients received corresponding treatment regimens at Renji Hospital from January 1^st^ 2019 to December 31^st^ 2021. Of the 30 patients, 10 patients received tislelizumab therapy with ongoing androgen deprivation therapy, the remaining 20 patients received docetaxel plus tislelizumab therapy with ongoing androgen deprivation therapy. Patients received 75 mg/m^2^ docetaxel intravenously every 3 weeks, 200 mg tislelizumab intravenously every 3 weeks. In the docetaxel plus tislelizumab group, the median number of docetaxel cycles was 5 (range: 2-8); the median number of tislelizumab cycles was 5 (range: 2-8). In the tislelizumab group, the median number of tislelizumab cycles was 2 (range: 1-4). The PSA and testosterone levels were evaluated every 2-4 weeks since initiation of treatment. The clinical characteristics and PSA follow-up information of the 30 patients was summarized in Table 1. Chi-square and Fisher's exact test was used to compare categorical variables and Wilcox rank sum test for continuous data. PSA progression- free survival data was analyzed by Kaplan-Meier survival curve. These data were collected under Renji Hospital Institutional Review Board.

### Statistical Analysis

All statistical and bioinformatic analyses were completed by the R software (v3.6.3) and GraphPad Prism software (v8.0). Graphics and plots were created by the ggplot2 R package and GraphPad Prism software (v8.0). Variance homogeneity was examined using the F-test. Then statistical analysis was performed using the t-test or the Wilcox rank sum test to compare the means between two unpaired groups with equal or unequal variance, respectively. For comparison of paired data, P values were calculated using paired t-test for parametric distributed paired data or Wilcoxon paired rank test for non-parametric distributed paired data. Kaplan-Meier survival analyses were completed using survival and survminer R packages with median value as the cut-off value to divided patients into two groups. Data were represented as mean values ± SEM, and significant P value was set at <0.05.

## Results

### Chemohormonal therapy activates immune response in prostate cancer

**Figure 1:**
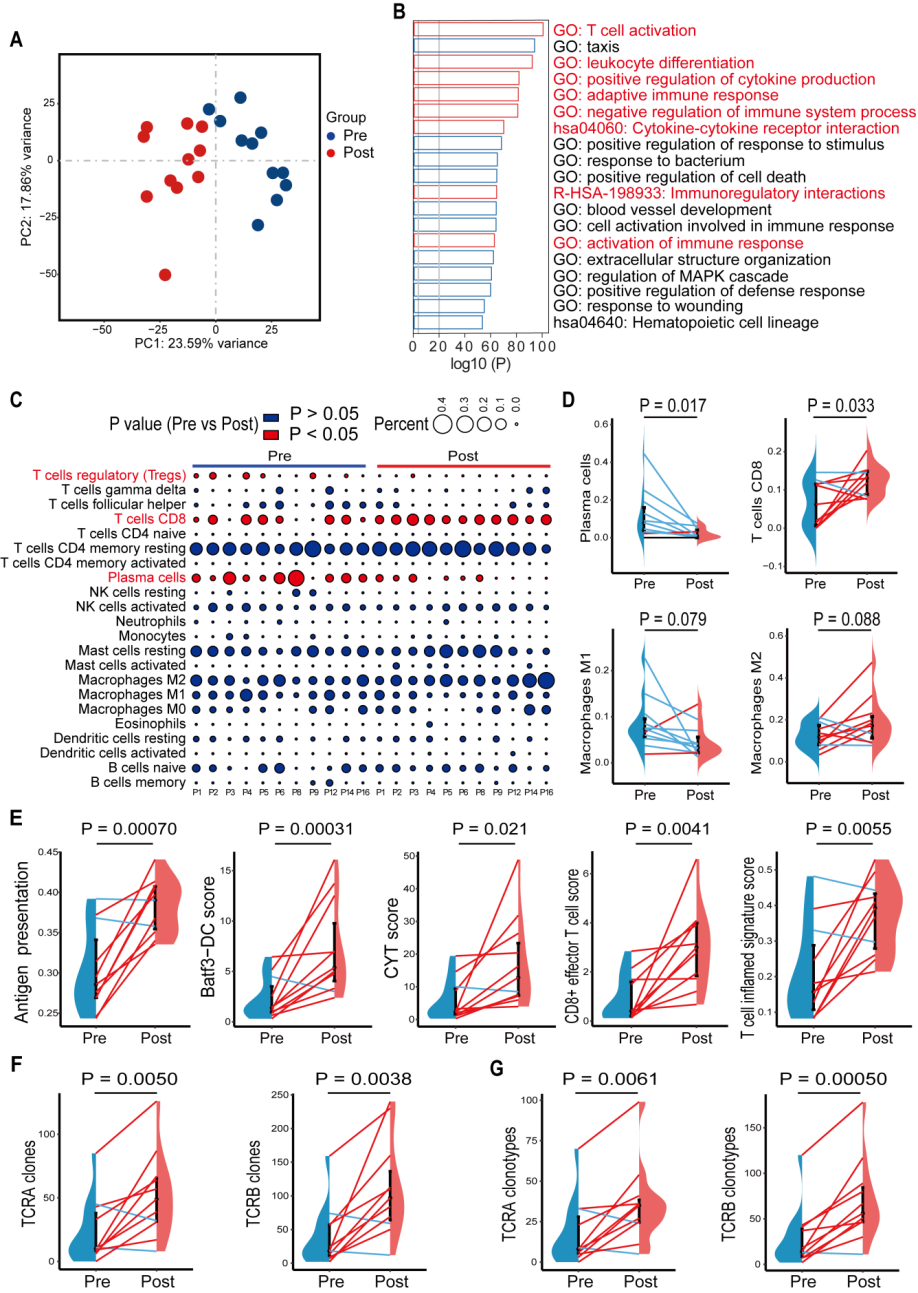
Gene signatures of tumors before and after chemohormonal therapy in prostate cancer patients. **A.** Principal component analysis of transcriptomic data from all 22 patient samples. Each dot represents a patient sample that is colored on the basis of treatment (blue, pretreatment; red, posttreatment). **B.** The horizontal bar graph showing the top 20 of upregulated differentially enriched pathways and functions in post-chemohormonal therapy tumor samples compared to paired pretreatment samples. Red bars indicate the immune-related differentially expressed pathways and functions. **C.** Bubble plot illustrating relevant immune cell profiles of paired pre- and post-chemohormonal therapy tumor samples. Bubble size reflects the percentage of immune cell subtypes enriched in corresponding immune cell profile. Bubble color reflects the P value. **D.** Changes in fractions of plasma cells, CD8 T cells, M1 macrophages, and M2 Macrophages between paired pre- and post-chemohormonal therapy tumor samples. **E.** Changes in antigen presentation score, Batf3-DC signature score, and three T cell-phenotype signatures scores (CYT score, CD8+ effector T cell score, and T cell inflamed signature score) between paired pre- and post-chemohormonal therapy tumor samples. **F.** Changes in the numbers of TRA and TRB clones detected between paired pre- and post-chemohormonal therapy tumor samples.** G.** Changes in the numbers of individual TRA and TRB clonotypes between paired pre- and post-chemohormonal therapy tumor samples. Each point represents an independent sample. Data were presented as mean values ± SEM. Paired data were analyzed using the paired t-test or Wilcoxon paired rank test.

In a previous study, we showed that high-risk locally advanced prostate cancer patients that received docetaxel-based neoadjuvant chemohormonal therapy had better biochemical progression-free survival time, compared with the other two controlled groups 10. This study prompted us to assess the response of the tumor microenvironment to docetaxel-based chemotherapy. We collected 11 paired pre- and post-chemohormonal therapy samples and performed RNA-sequencing (Table S2). Principal Component Analysis revealed that samples with chemohormonal therapy are transcriptionally distinct from paired pretreatment samples (Figure 1A). Using differential expression analysis, we observed chemohormonal therapy significantly modulated the expression of 5265 genes, including 3690 upregulated and 1575 downregulated genes (Figure S1A). Notably, 32.2% of the up-regulated genes and 16.9% of the down-regulated genes were immune-related (Figure S1B). Further analysis of the 3690 upregulated genes revealed significant enrichments in immune-related pathways (Figure 1B). In addition, T cell, B cell, and NK cell-mediated immunity were strengthened after chemohormonal therapy (Figure S1C). To study in more detail which immune cell subtypes may be associated with these changes, we applied CIBERSORT to characterize 22 inferred immune subsets for the 11 paired samples and found that post-chemohormonal therapy samples were significantly associated with increased CD8 T cells and decreased regulatory T cells (Figure 1C-D), while the densities of B cells, NK cells, and macrophages were not statistically different between paired samples (Figure 1C-D and Figure S1E). Consistently, T cell antitumoral activity-related metrics, including antigen presentation score, Batf3-dendritic cell (DC) signature score, CYT score, CD8^+^ effector T cell score, and T cell inflamed score, were increased in post-chemohormonal therapy samples (Figure 1E). Two gene sets contributing to T cell activation - interferon gamma and TNFα signaling pathway - were also significantly enriched in post-chemohormonal therapy samples (Figure S1D). Using MiXCR, we detected higher numbers of both TCRA and TCRB clones and specific clonotypes in post-chemohormonal therapy samples (Figure 1F-G). In addition, post-chemohormonal therapy samples tended to have a higher level of immune checkpoints, such as PD1, PD-L1, CTLA4, TIGIT, and LAG3 (Figure S1F). All these transcriptome results indicated that chemohormonal therapy may induce the T cell-mediated immune response in the tumor microenvironment.

### Hormone therapy shows no significant impact on the immune response

To determine which treatment contributes to immune activation in the tumor microenvironment, we extended a previously published RNA-seq dataset - GSE111177, which contains 20 paired pre- and post-hormonal therapy prostate cancer tissues 14. Although Principal Component Analysis showed transcriptome profiles before and after hormonal therapy were distinguished (Figure S2A), Gene Set Enrichment Analysis (GSEA) indicated that the immune response gene set was not enriched in post-hormonal therapy samples (Figure S2B). Moreover, T cell antitumoral activity-related metrics showed no significant difference between pre- and post-hormonal therapy samples (Figure S2C). Although the two gene sets contributing to T cell activation were enriched in post-chemohormonal therapy samples, they showed no difference in enrichment between pre- and post-hormonal therapy samples (Figure S2D). These data suggested hormone therapy may not be the major role that activates immune responses in prostate cancer.

### Assessment of T cells in tumor microenvironment before and after chemohormonal therapy

**Figure 2:**
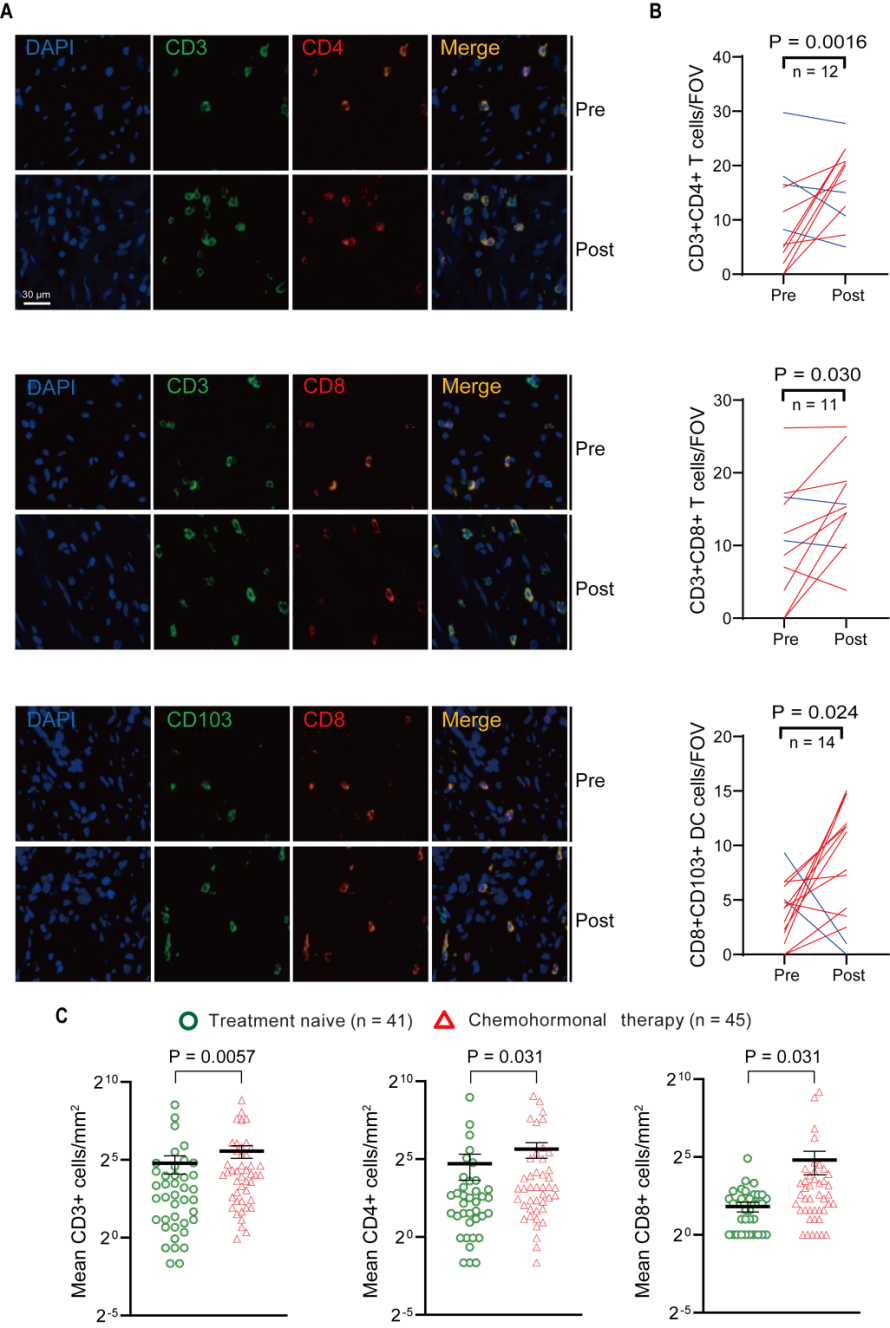
Multiplex immunofluorescence and immunohistochemistry assessment of immune cells in the tumor microenvironment of pre- and post-chemohormonal therapy tumor samples.** A.** Representative fluorescence images of immunolabeled CD4^+^ T cells (CD4^+^CD3^+^), CD8^+^ T cells (CD8^+^CD3^+^), and tumor-resident T cells (CD103^+^CD8^+^) from paired pre- and post-chemohormonal therapy tumor samples. **B.** Changes in the densities of immune cells in (A) between paired pre- and post-chemohormonal therapy tumor samples. FOV, field of view.** C.** Scatterplots comparing the densities of tumor-infiltrating lymphocytes, including CD3^+^ cells, CD4^+^ cells, and CD8^+^ cells between treatment naive and chemohormonal therapy treated tumor samples. Each point represents an independent sample. Data were presented as mean values ±SEM. Unpaired data were analyzed using the t-test or Wilcox rank sum test. Paired data were analyzed using the paired t-test or Wilcoxon paired rank test.

Above transcriptome analysis results prompted us to focus on the immune remodeling in the microenvironment after chemohormonal therapy, especially the enrichment of tumor-infiltrating T cells. We collected matched pre- and post-chemohormonal therapy samples from prostate cancer patients and performed multiplex immunofluorescent staining to assess intratumoral immune cell composition. Quantitative analysis revealed that chemohormonal therapy significantly increases the intratumoral infiltration of CD3^+^CD4^+^ T cells, CD3^+^CD8^+^ T cells, and CD103^+^CD8^+^ tumor-resident T cells (Figure 2A-B).

Next, the density of intratumoral T cells was further analyzed by immunohistochemistry in tissue microarrays from another prostate cancer cohort (Table S1), including 41 treatment-naive samples and 45 post-chemohormonal therapy samples. Compared with treatment-naive group, chemohormonal therapy group had a higher density of CD3^+^, CD4^+^, and CD8^+^ cells (Figure 2C and Figure S3A). Together, these results strongly confirmed that docetaxel-based chemohormonal therapy could induce T cell-mediated immune responses in prostate cancer patients.

We also assessed the tumor-infiltrating macrophages (CD163^+^CD68^+^) in tumor samples (Figure S3B). The median number of overall macrophages in both groups remained very low, although post-chemohormonal therapy samples showed a slightly increased number of macrophages, compared with paired pretreatment controls (Figure S3C). In addition, CD20^+^ B cells and CD56^+^ NK cells did not change significantly after chemohormonal therapy (Figure S3D-G).

### Chemohormonal therapy enhances immune response through the cGAS/STING pathway

We next sought to determine how chemohormonal therapy modulates the prostate cancer microenvironment. Mining of datasets from paired pre- and post-chemohormonal therapy samples revealed the enrichment of DNA damage and type I IFN pathway after chemohormonal therapy (Figure S4A). We also observed the activation of the cGAS/STING pathway in post-chemohormonal therapy samples, compared with paired pre-treatment tissues (Figure S5A). In contrast, hormonal treatment alone induced neither the cGAS/STING pathway nor DNA damage and the IFN pathway (Figure S4B-C), suggesting again the critical role of docetaxel-based chemotherapy in this progression.

Given that cGAS serves as a cytosolic DNA sensor and induces STING activation, subsequently upregulating IFN-stimulated genes and promoting T cell infiltration 16, 17, we therefore hypothesized that docetaxel-based chemohormonal therapy may recruit T cells through the cGAS/STING-IFN pathway. We first confirmed that tumor tissues with chemohormonal treatment showed a higher level of IFN-stimulated genes, such as IFIT1, IFI44, CCL5, and IFNB, compared with treatment-naive tissues (Figure S5B).

**Figure 3:**
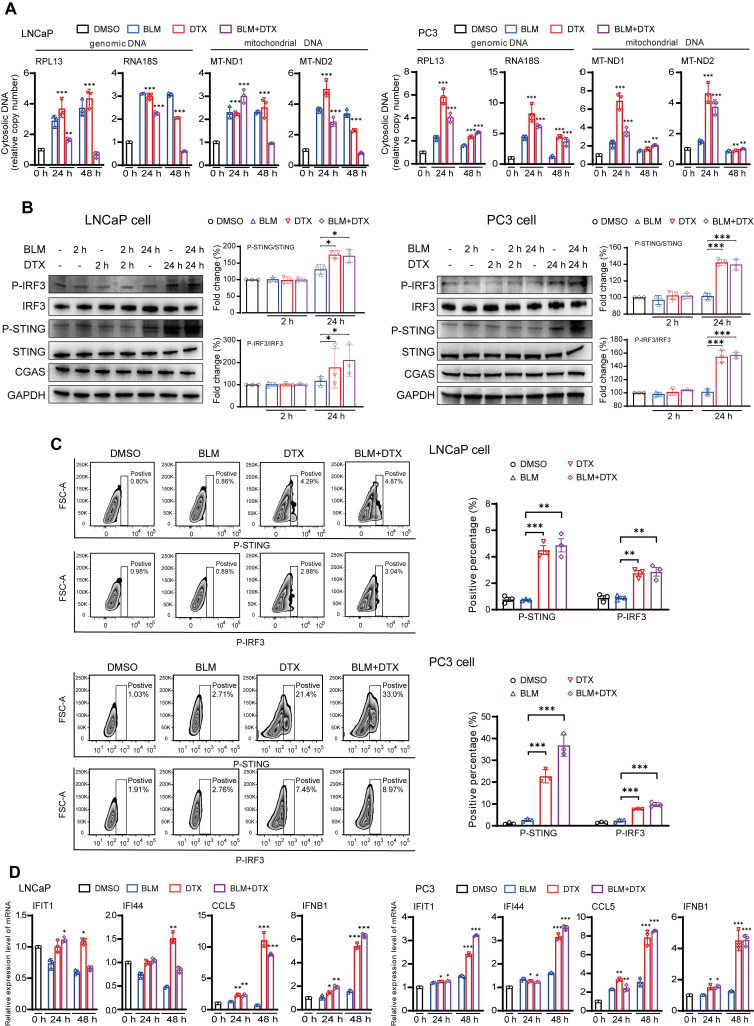
Combination of docetaxel and androgen-deprived treatment activates the cGAS/STING pathway in prostate cancer cells. **A.** QRT-PCR analysis of genomic DNA (RPL13 and RNA18S) and mitochondrial DNA (MT-ND1 and MT-ND2) in LNCaP cells (left panel) and PC3 cells (right panel) after treatment with DMSO, bicalutamide (BLM), docetaxel (DTX), or bicalutamide plus docetaxel (BLM+DTX) for the indicated time. **B.** Western blot analysis of cGAS/STING pathways components with indicated antibodies in LNCaP cells (left panel) and PC3 cells (right panel) after treatment with DMSO, bicalutamide (BLM), docetaxel (DTX), or bicalutamide plus docetaxel (BLM+DTX) for the indicated time. **C.** Representative flow plots and quantification of the positive percentage of p-STING and p-IRF3 expression in LNCaP cells (upper panel) and PC3 cells (lower panel) after DMSO, bicalutamide (BLM), docetaxel (DTX), or bicalutamide plus docetaxel (BLM+DTX) treatment for 24 hours. **D.** QRT-PCR analysis of cGAS/STING pathway downstream immune genes in LNCaP cells (left panel) and PC3 cells (right panel) after treatment with DMSO, bicalutamide (BLM), docetaxel (DTX), or bicalutamide plus docetaxel (BLM+DTX) for the indicated time. Each point represents an independent experiment. Data were presented as mean values ± SEM. Unpaired data were analyzed using the t-test or Wilcox rank sum test. *, P < 0.05; **, P < 0.01; ***, P < 0.001.

We next used an* in vitro* cell line system, consisted of castration-sensitive (LNCaP and LAPC4) and castration-resistant prostate cancer cell lines (PC3, DU145, and 22Rv1), to aid in dissecting the complex cascades in the tumor microenvironment after docetaxel-based chemohormonal therapy. We treated the cells with docetaxel with or without bicalutamide and analyzed the purified cytosolic extracts. Both genomic and mitochondrial DNA fragments were released into the cytosol after treatment (Figure 3A). Specially, Western blot and flow cytometry data showed that docetaxel and combined treatment activated the cGAS/STING pathway stronger than bicalutamide alone (Figure 3B-C). Then we asked whether this activation can induce IFN-stimulated genes, which may elicit T cells infiltration to the tumor microenvironment. Among the cell lines, DU145 that showed a higher expression level of STING is correlated with a higher level of IFN-stimulated genes. In contrast, since endogenous STING was undetectable in 22Rv1 cells, we could not detect IFN-stimulated genes expression in this cell line (Figure S5C). As expected, docetaxel with or without bicalutamide treatment significantly upregulated IFN-stimulated genes in several cell lines (Figure 3D and Figure S5D-E). We generated STING knockout LNCaP cells and STING knockdown RM1 cells and showed that STING deficiency could attenuate the upregulation of the IFN signaling induced by docetaxel treatment (Figure S5F and Figure S7A, C). Altogether, these data indicated docetaxel-based chemohormonal therapy can trigger DNA release into the cytosol, thereby activating the cGAS/STING pathway, which leads to IFN-stimulated genes response in prostate cancer cells. Of note, docetaxel alone is sufficient to trigger cytosolic DNA-cGAS/STING-IFN axis, more significantly than bicalutamide alone.

Additionally, by mining the prostate cancer dataset from TCGA, we observed a direct correlation between high cGAS expression and better survival, suggesting that cGAS could play a protective role in prostate cancer patient prognosis (Figure S6A). Moreover, IFN-stimulated genes are significantly associated with CD8A and CD3D expression, indicating the correlation between the IFN signaling and T cells infiltration in prostate cancer samples (Figure S6B).

### Chemohormonal therapy synergizes with anti-PD1 blockade to inhibit tumor growth in murine prostate cancer models

**Figure 4:**
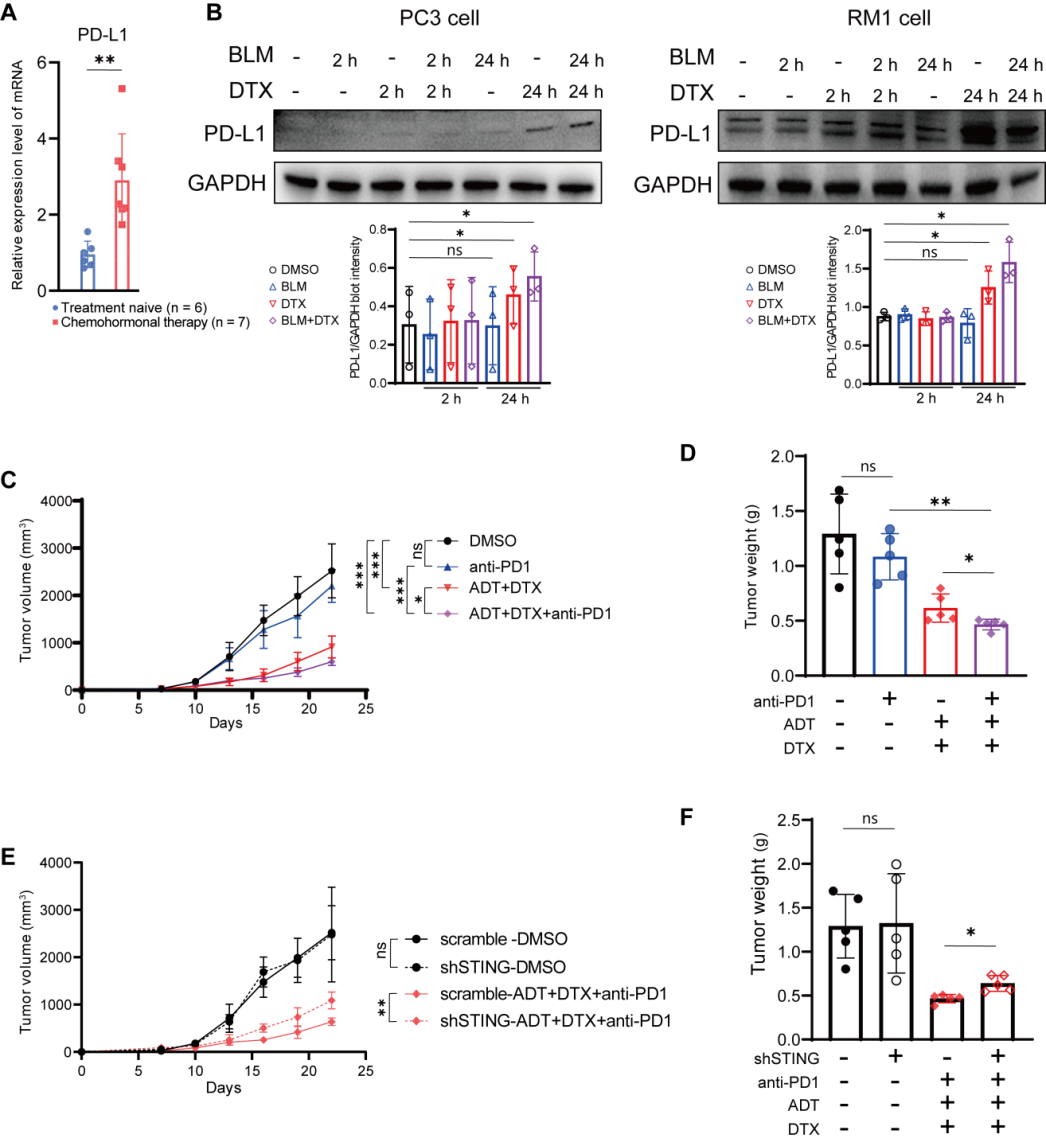
Chemohormonal therapy sensitizes RM1 tumor-bearing mice to anti-PD1 blockade therapy. **A.** QRT-PCR analysis of PD-L1 mRNA level in treatment naive and chemohormonal therapy tumor samples. Each point represents an independent sample. **B.** Western blot showing the protein level of PD-L1 in PC3 cells (left panel) and RM1 cells (right panel) after treatment with DMSO, bicalutamide (BLM), docetaxel (DTX), or bicalutamide plus docetaxel (BLM+DTX) for the indicated time. Each point represents an independent experiment.** C-D.** Tumor size (C) and tumor weight (D) of RM1 tumor xenografts in C57 mice, treated with castration plus either DMSO, anti-PD1 (RMP1-14, 6 mg/kg), bicalutamide (20 mg/kg) + docetaxel (10 mg/kg), or combination treatment. Tumor sizes were measured every 3 days. Each point represents an independent sample. **E-F.** Tumor size (E) and tumor weight (F) of RM1 (scramble or shSTING) tumor xenografts in C57 mice, treated with castration plus either DMSO or anti-PD1 (RMP1-14, 6 mg/kg) + bicalutamide (20 mg/kg) + docetaxel (10 mg/kg). Tumor sizes were measured every 3 days. Each point represents an independent sample. Data were presented as mean values ±SEM. Unpaired data were analyzed using the t-test or Wilcox rank sum test. *, P < 0.05; **, P < 0.01; ***, P < 0.001.

Based on above results, we hypothesized that docetaxel-based chemohormonal therapy can improve the efficacy of immune therapy. Therefore, we first examined prostate cancer samples and found that docetaxel-based chemohormonal therapy elevated the PD-L1 expression, compared with treatment-naive group (Figure 4A). *In vitro* cell culture experiments also confirmed that docetaxel with or without bicalutamide could significantly elevate PD-L1 protein level (Figure 4B). To examine the antitumor efficacy of combination treatment in immune-competent mice, we generated subcutaneous xenografted RM1 tumors, which were considered as androgen-insensitive prostate cancer, in castrated male C57/BL6 mice and treated xenografted tumors with DMSO, anti-PD1 blockade, bicalutamide/docetaxel, and anti-PD1 blockade/bicalutamide/docetaxel. Although bicalutamide plus docetaxel are sufficient to inhibit the tumor growth, addition of anti-PD1 blockade exerted a strongly synergistic inhibitory effect on both tumor volume and tumor weight (Figure 4C-D).

We further confirmed the function of the cGAS/STING pathway in the antitumor immune response on the xenograft mouse model. RM1 cells containing shRNA targeting STING showed no statistical difference in cell viability and tumor growth rate in the absence of treatment, compared with control shRNA (Figure S7A-B and Figure 4E-F). In contrast, RM1 STING shRNA-bearing mouse model showed less response to anti-PD1 blockade/bicalutamide/docetaxel treatment (Figure 4E-F). Consistently, RM1 cells with STING shRNA showed less activation of IFN-stimulated genes after bicalutamide and docetaxel treatment, compared with control shRNA (Figure S7C). All these results strengthen our hypothesis that the cGAS/STING pathway serves as a functional effector, at least partially, mediating the growth inhibition induced by combined therapy in prostate cancer.

### Chemohormonal therapy activates immune response to suppress xenografted tumors growth

**Figure 5:**
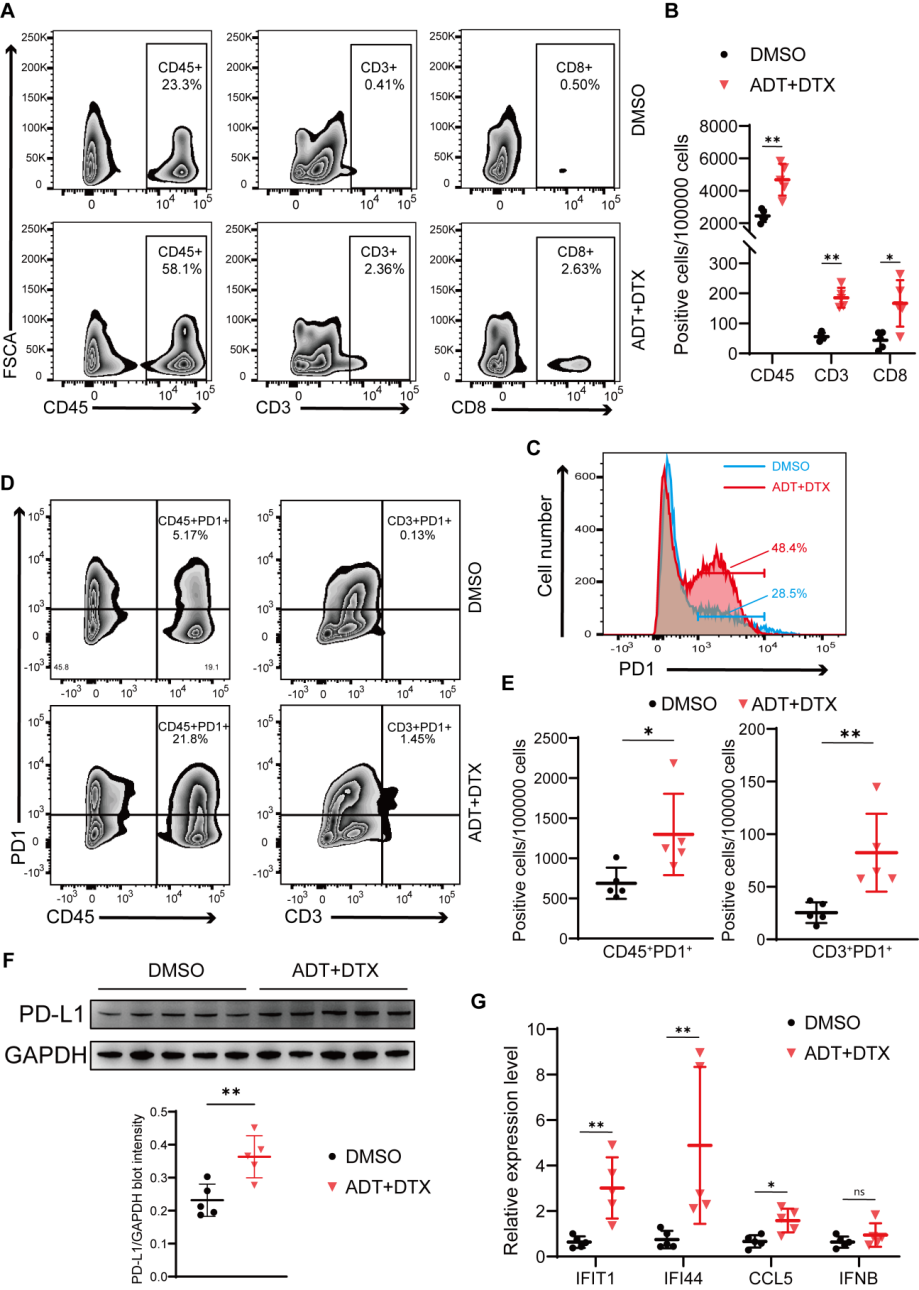
Chemohormonal therapy activates immune response to suppress xenografted tumors growth. **A-B.** Representative flow plots (A) and densities (B) of intratumoral CD45^+^ leukocytes, CD3^+^ T cells, and CD8^+^ T cells in xenografted tumors of indicated groups. **C.** The distribution of PD1 expression in xenografted tumors of indicated groups. The proportion of cells is indicated in the plot. **D-E.** Representative flow plots (D) and densities (E) of CD45^+^PD1^+^ cells and CD3^+^ PD1^+^ cells in xenografted tumors of indicated groups. **F.** Western blot showing the protein level of PD-L1 in xenografted tumors of indicated groups. **G.** QRT-PCR analysis of cGAS/STING pathway downstream immune genes in xenografted tumors of indicated groups. Each point represents an independent sample. Data were presented as mean values ± SEM. Unpaired data were analyzed using the t-test or Wilcox rank sum test. *, P < 0.05; **, P < 0.01; ***, P < 0.001.

To test the effects of docetaxel-based chemohormonal therapy on T cell-mediated immune response in the tumor microenvironment, we collected xenografted tumors from above mouse models and analyzed T cell infiltration within the tumors by flow cytometry and immunohistochemistry staining. As anticipated, chemohormonal treatment triggered a significant increase of intratumoral CD45^+^ leukocytes (Figure 5A-B). Detailed cell type analysis revealed that CD3^+^ and CD8^+^ T cells were increased within tumors after chemohormonal treatment (Figure 5A-B and Figure S8A). Notably, compared with DMSO treatment, xenografted tumors exhibited higher PD1 abundance after chemohormonal treatment, especially for intratumoral CD45^+^ leukocytes and CD3^+^ T cells (Figure 5C-E). We also observed that PD-L1 protein level was increased in xenografted tumors after chemohormonal treatment (Figure 5F), which is consistent with the upregulation of PD-L1 abundance in patient samples and *in vitro* prostate cancer cell lines after bicalutamide plus docetaxel treatment (Figure 4A-B). Docetaxel with bicalutamide treatment significantly upregulated IFN-stimulated genes in xenografted tumors (Figure 5G), again consistent with results in prostate cancer cell lines (Figure 4D and Figure S5D-E).

In line with the above result that STING knockdown attenuated the tumor growth inhibition effect of bicalutamide/docetaxel/anti-PD1 blockade on the xenograft mouse model, the percentage of CD3^+^ and CD8^+^ T cells was decreased in RM1 STING shRNA-bearing tumor samples after combined treatment, compared with control shRNA, although the percentage of T cells showed no difference in treatment-naive tumors between STING shRNA and control shRNA (Figure S8B-C). These results again confirmed that the cGAS/STING-IFN axis induced T cell infiltration partially mediated the tumor growth inhibition effect induced by combination therapy in prostate cancer.

### Pilot clinical study to determine mCRPC response to docetaxel plus tislelizumab therapy

**Figure 6:**
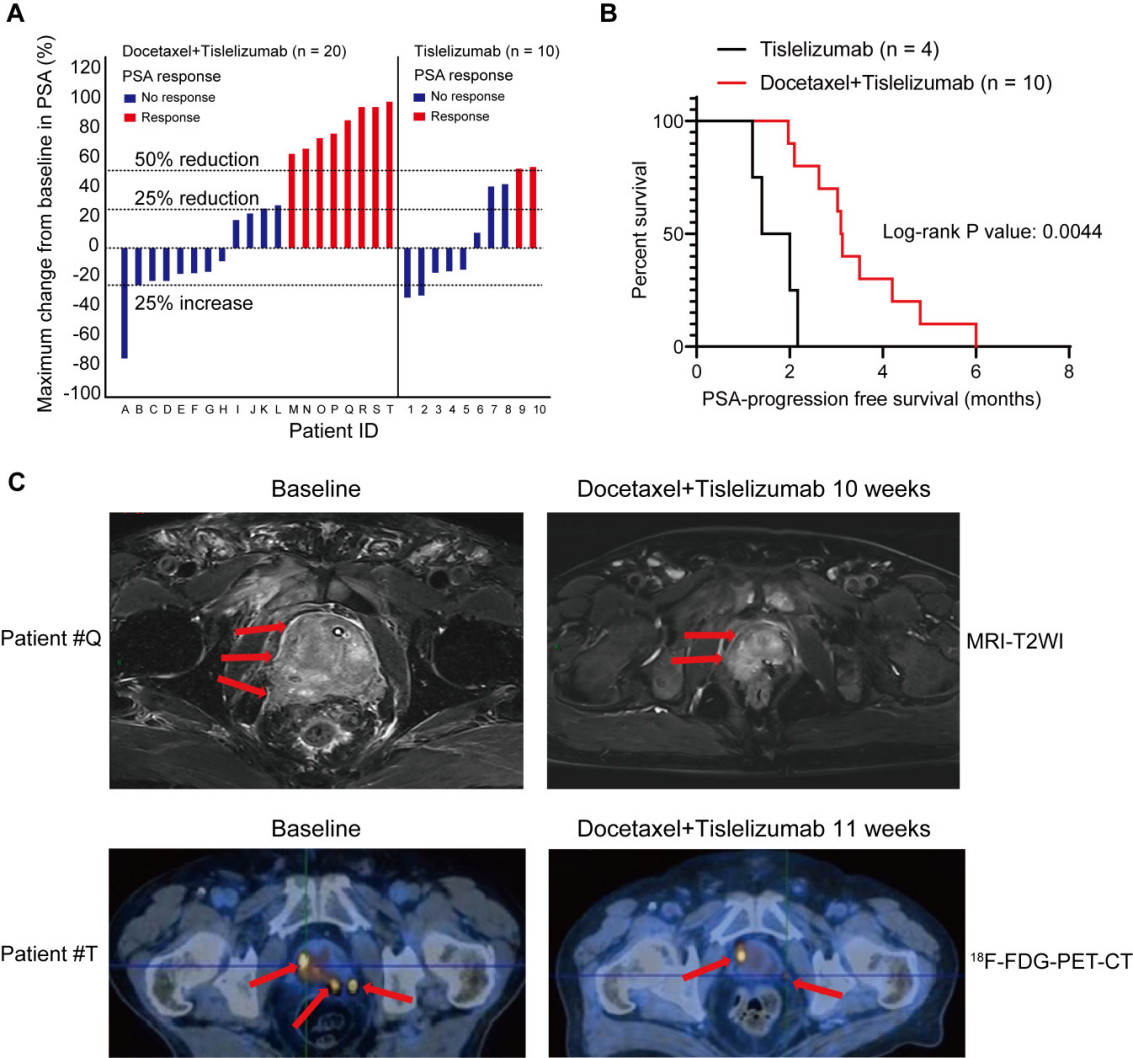
Pilot clinical study to determine metastatic castration-resistant prostate cancer (mCRPC) response to docetaxel plus tislelizumab therapy. **A.** PSA responses among chemotherapy-naive mCRPC patients after the treatment of tislelizumab with or without docetaxel. Patients were labelled from A to T (left panel) or 1-10 (right panel). **B.** PSA progression-free survival in patients with ≥25% PSA reduction after the treatment of tislelizumab with or without docetaxel. **C.** The representative MRI images (upper panel) and ^18^F-FDG-PET-CT images (lower panel) showing tumor regression in two patients (patient #Q and #T) during the combination treatment of docetaxel and tislelizumab.

To evaluate the antitumor efficacy of the combined treatment of docetaxel-based chemotherapy and immune checkpoint inhibitor in human prostate cancer, we performed a retrospective analysis for 30 mCRPC patients, who failed prior abiraterone treatment and did not receive any chemotherapy before. 10 patients from this analysis were treated with tislelizumab, an anti-human programmed death receptor monoclonal IgG4 antibody; the remaining 20 patients were treated with tislelizumab plus docetaxel. Table 1 showed comparable baseline characteristics of the two treatment groups including age, visceral metastases rate, and baseline PSA level. After combined treatment of docetaxel and tislelizumab, 40% patients (8 of 20) achieved a PSA response (defined as a ≥50% PSA reduction), 50% patients (10 of 20) had a ≥25% reduction of PSA level from baseline (Figure 6A and Table 1). In contrast, only 20% (2 of 10) patients from tislelizumab group achieved a PSA response and 40% (4 of 10) had a ≥25% PSA reduction (Figure 6A and Table 1). However, these results did not reach statistical significance, most likely due to the relatively small size of our cohort (Table 1). Notably, for patients with a ≥25% PSA reduction, a better median PSA progression-free survival was seen in the docetaxel plus tislelizumab group (3.12 months (95% CI: 2.56-4.34) vs. 1.70 months (95% CI: 0.95-2.43), p = 0.0044) (Figure 6B). Representative radiographs of 2 patients before and after the treatment of docetaxel and tislelizumab showed evident regression of primary tumors, suggesting the partial response (Figure 6C). These early clinical data support our hypothesis that docetaxel synergizes with anti-PD1 checkpoint inhibitor immunotherapy, which may benefit mCRPC patients.

## Discussion

In this study, we investigated the effect of docetaxel-based chemohormonal therapy on the prostate cancer microenvironment and evaluated whether this treatment can trigger antitumoral immune responses in the disease. Our results suggest that docetaxel combined with hormonal therapy effectively boosted T cell-mediated immune responses, supported by the following findings: 1) Transcriptome profiling analysis revealed that T cell antitumoral activity-related signatures were upregulated after chemohormonal therapy; 2) Histopathology staining confirmed the increased number of infiltrated T cells on human prostate cancer sites after chemohormonal therapy; 3) Murine models showed docetaxel combined with androgen-deprived therapy enhances T cell infiltration into the tumor microenvironment. Importantly, analyses of GSE111177 revealed that hormonal therapy alone could not activate T cell-related immune responses in prostate cancer samples (Figure S2) 14. Moreover, in the cell culture system, docetaxel alone is sufficient to trigger cytosolic DNA-cGAS/STING-IFN axis, more significantly than bicalutamide alone (Figure 3). Other studies have reported that androgen receptor antagonists, including flutamide and enzalutamide, enhanced myeloid cell-mediated immune suppression and impaired the T cell-mediated antitumoral activity 18, 19. All these results lead to the conclusion that docetaxel is the dominant factor for immune activation in prostate cancer. It is worth noting that docetaxel treatment has a dual effect of prompting T cell infiltration and upregulating PD1 and PD-L1 abundance. Docetaxel-based chemohormonal therapy upregulated PD-L1 abundance in both patient samples and xenografted tumors (Figure 4A and Figure 5F). This observation was confirmed by experiments on prostate cancer cell lines that docetaxel treatment increased PD-L1 protein level in PC3 and RM1 cells (Figure 4B). By flow cytometry, we also observed that chemohormonal therapy upregulated PD1 abundance in tumor-infiltrating lymphocytes (Figure 5D-E). Above data indicated a pleiotropic effect of docetaxel on not only prostate cancer cells but also the tumor microenvironment by increasing the infiltration of T cells and upregulating the abundance of PD1 and PD-L1 abundance. Therefore, it is reasonable to assume that chemohormonal therapy combined with anti-PD1 blockade could improve antitumor therapy, which we will discuss later.

Docetaxel treatment could cause mitochondrial and genomic DNA release in cancer cells, as a consequence of cell apoptosis 20. cGAS is a cytosolic DNA sensor, which activates the downstream adaptor STING, recruits the transcription factor IRF3 into the nuclear via a phosphorylation-dependent mechanism. Activation of the cGAS/STING-IRF3 pathway can induce interferon-related genes such as IFIT1, IFI44, CCL5, and IFNB, which may promote T cell mobilization for antitumor immunity 16, 17, 21. In our study, the transcriptome analysis indicates that docetaxel combined with androgen-deprived treatment activates the cGAS/STING pathway and downstream interferon-stimulated genes. In tumor tissues, we confirm this activation and show a significant association of interferon-stimulated genes mRNA upregulation with chemohormonal therapy, compared with treatment-naive samples (Figure 3B). Our *in vitro* experiments suggest that docetaxel treatment drives cytoplasmic accumulation of genomic and mitochondrial DNA, which is sensed by the cGAS/STING pathway (Figure 3C). Interestingly, in 22Rv1 cells, which lack endogenous STING, the expression of interferon-stimulated genes was hardly detected (Figure S5A). Moreover, knockout of STING in LNCaP cells reduced the elevation of interferon stimulated genes under bicalutamide or docetaxel treatment (Figure S5E). In a murine model, knockdown of STING not only restricted the efficacy of anti-PD1 plus chemohormonal therapy but also decrease T cell infiltration in the tumor microenvironment (Figure 4E-F). Altogether, these findings suggest that the cGAS/STING-IFN pathway plays an important role in mediating antitumor immunity. Our study does not exclude other pathways that may also be involved in this complex cascade. For instance, several recent articles highlighted the role of docetaxel-induced cellular senescence in the progression of tumor therapy 22. Senescent cells produce a number of cytokines and chemokines, so-called senescence-associated secretory phenotype, and modulate themselves as well as their neighbor immune cells, suggesting another possible link between docetaxel and immune responses 16, 23. We also do not exclude the effect of docetaxel on other cells in the microenvironment, such as immune cells themselves. A previous study reported that the cGAS/STING pathway in dendritic cells mediated sensing of irradiated-tumor cells and enhanced adaptive immune responses to radiation 24. Another group showed that the cGAS/STING pathway in tumor cells contributes to T cell priming, and sensitizes tumor to checkpoint therapy 25. Our findings add a new dimension to understanding this critical pathway in the crosstalk between tumor cells and the microenvironment.

The upregulation of antitumoral immune response and PD1/PD-L1 expression by chemotherapy prompted us to explore the possibility of combining docetaxel with anti-PD1 blockade in treating prostate cancer, especially in treating CRPC patients. To verify the hypothesis, we used a xenograft mouse model and a mCRPC patient cohort. We used RM1 cells, which are considered as androgen insensitive cell lines 26, to establish the xenograft mouse model and assess the efficacy of the combination treatment. The results confirmed that docetaxel-based chemohormonal therapy synergized with anti-PD1 blockade, although this xenograft mouse model still responded to androgen deprivation plus docetaxel (Figure 4 and Figure S7). Similar encouraging results came from a retrospective analysis of 30 mCRPC patients who progressed on abiraterone. The addition of docetaxel to tislelizumab has effectively improved the PSA progression-free survival of mCRPC patients (Figure 6B). Limitations of our study include the lack of overall survival data and the small size of the cohort. Several prospective trials on a larger scale evaluating the efficacy of immune checkpoint blockade combination therapy for mCRPC patients are ongoing (NCT02861573 and NCT03338790).

In summary, our study demonstrated that docetaxel-based chemohormonal therapy can reprogram tumor microenvironment in a manner that propagates T cell immunity by augmenting the DNA-cGAS/STING-IFN signaling. These findings not only extend the understanding of the pleiotropic effects of docetaxel on tumor cells and the microenvironment, but also provide a rational cooperative strategy that has the potential clinical translational value in efficient immune therapy of prostate cancer.

## Supplementary Material

Supplementary methods, figures and tables.

## Figures and Tables

**Table 1:** Clinical characteristics and treatment responses in patients collected for this retrospective analysis

Characteristics	Category	Docetaxel+Tislelizumab (n = 20)	Tislelizumab (n = 10)	P value
Age, years	Median (range)	72 (56-84)	73 (64-82)	0.58
Visceral metastases, %	Percentage	10	10	1.00
Baseline PSA level, ng/mL	Median (range)	54.70 (5.04-2768.00)	150.00 (23.00-310.00)	0.082
PSA response rate, %	Percentage (95% CI)	40 (18.53-61.47)	20 (<0-44.79)	0.42
Time to PSA progression, months	Median (95% CI)	3.12 (2.56-4.34)	1.70 (0.95-2.43)	0.0044

PSA: prostate-specific antigen; CI: confidence interval.

## Abbreviations

PSA: Prostate-specific antigen
TCGA: The Cancer Genome Atlas Program
GSEA: Gene Set Enrichment Analysis
ssGSEA: Single-sample Gene Set Enrichment Analysis
DC: Dendritic cell
ADT: Androgen-deprived treatment
mCRPC: metastatic castration-resistant prostate cancer
CI: Confidence interval
