# Supplementary material for: Docetaxel remodels prostate cancer immune microenvironment and enhances checkpoint inhibitor-based immunotherapy
Source: Theranostics. 2022 Jun 27;12(11):4965–79. doi: 10.7150/thno.73152 (PMC9274752; doi:10.7150/thno.73152)
Supplement: Supplementary file 1 — Supplementary methods, figures and tables. [file thnov12p4965s1.pdf]

Supplementary materials for:

Docetaxel remodels prostate cancer immune microenvironment and enhances checkpoint inhibitor-based immunotherapy

Zehua Ma, Weiwei Zhang, Baijun Dong, Zhixiang Xin, Yiyi Ji, Ruopeng Su, Kai Shen, Jiahua Pan, Qi Wang, Wei Xue

Supplementary methods

Immunohistochemistry staining

For immunohistochemistry staining of CD20 (1:100; MXB Biotechnologies; MAB-0669) and CD56 (1:100; MXB Biotechnologies; MAB-0743), 3um paraffin-embedded sections were stained. For immunohistochemistry staining of CD3 (1:200; Abcam; ab16669), CD4 (1:100; MXB Biotechnologies; RMA-0620), and CD8 (1:100; MXB Biotechnologies; RMA-0514), tissue microarrays were stained. The paraffin-embedded sections or tissue microarrays were unmasked in 1 × Tris-EDTA buffer (pH 9.0) for 20 minutes at 95 °C and then incubated with specific antibodies for overnight at 4 °C. For paraffin-embedded sections, digitalized images were taken using Nikon-80i microscope under 40× objective. For tissue microarray, slides were scanned using Leica Aperio AT2 under 40× objective.

For quantification of CD20 and CD56, two independent researchers calculated the average number of membrane-positive cells in five to six random 40× fields. For quantification of CD3, CD4 and CD8, two independent researchers considered the whole filed and evaluated number of membrane-positive cells.

Immunofluorescence staining

For staining of intratumoral immune cell subsets, we used CD3 (1:400; Abcam; ab16669) as pan-T cell marker, CD4 (1:200; MXB Biotechnologies; RMA-0620) as T helper cell marker, CD8 (1:200; MXB Biotechnologies; RMA-0514) as cytotoxic T cell marker, CD68 (1:100; Abcam; ab955) as pan-macrophage marker and CD163 (1:500; Abcam; ab182422) as M2 macrophage marker. Tumor-resident T cells were immunolabelled by CD8 (1:200; MXB Biotechnologies; RMA-0514) and CD103 (1:500; Abcam; ab129202). Double immunofluorescence staining was performed in 3um paraffin-embedded sections using the Alexa Fluor™ 555 Tyramide SuperBoost™ Kit (Thermo Fisher; B40913) and Alexa Fluor™ 488 Tyramide SuperBoost™ Kit (Thermo Fisher; B40922) according to the provided manufacturer's instructions. Digitalized images were taken using Nikon-80i microscope under 40× objective. For quantification of immunofluorescence staining, two independent researchers calculated the average number of membrane-positive cells in five to six random 40× fields.

Transcriptome data analysis

Differential expression analysis was performed using the DESeq (2012) R

package. False discovery rate (FDR) value < 0.05 and foldchange > 2 or foldchange < 0.5 were set as the threshold for significantly differential expression. We used the Immunology Database and Analysis Portal database to identify immune-related differentially expressed genes (DEGs). Functional enrichment analysis of up-regulated DEGs was performed using the Metascape online tool (http://metascape.org).

Gene Set Enrichment Analysis (GSEA) was performed using the GSEA software (Version: 3.0; http://software.broadinstitute.org/gsea/index.jsp). The annotated gene set file (msigdb.v7.2.symbols.gmt) was selected as the reference gene set, and significance set at FDR < 0.25. The size of gene sets was set to 5-500 genes, with 1000 permutations.

Single-sample Gene Set Enrichment Analysis (ssGSEA) algorithm was performed using the gsva R package. The Gaussian distribution was chosen as kcdf argument, with a minimum and maximum geneset size of 5 and 500, respectively. Antigen presentation score, CD8+ effector T cell score, and T cell inflamed score were defined as the ssGSEA scores of relevant gene sets. Batf3-dendritic cell (DC) score was defined as the mean expression levels of genes included in the relevant gene set. CYT score was defined as the geometric mean of expression levels of GZMA and PRF1 (as expressed in TPM). The gene sets associated with the above scores were described in Supplemental Table S3.

We used CIBERSORT to estimate the relative fractions of intratumoral immune cell subsets according to the gene expression profiles. The LM22 file, which is a leukocyte gene signature matrix consisting of 547 genes, was used to define 22 immune cell types. The sum of fractions of all 22 intratumoral immune cell subsets is equal to 1 in each sample.

We used MiXCR to extract TCR and BCR CDR3 repertoires from RNA-Seq data.

QRT-PCR from Cell Lines

RNA isolation was performed using Trizol® method according to the manufacturer’s instructions (ThermoFisher; 15596026). RNA concentration and quality were evaluated using a NanoDrop apparatus (NaNodrop Technologies). cDNA was synthesized using HiScript® III RT SuperMix for qPCR kit (Vazyme; 7E402G0). QRT-PCR was performed using ChamQ Universal SYBR qPCR Master Mix (Vazyme; 7E472E0). Human QRT-PCR primer sequences were: GAPDH: 5’-GGAGCGAGATCCCTCCAAAAT-3’; 3’-GGCTGTTGTCATACTTCTCATGG-5’. IFIT1: 5’-GCCTTGCTGAAGTGTGGAGGAA-3’; 3’-ATCCAGGCGATAGGCAGAGATC-5’. IFI44: 5’-GTGAGGTCTGTTTTCCAAGGGC-3’; 3’-CGGCAGGTATTTGCCATCTTTCC-5’. CCL5: 5’-CCAGCAGTCGTCTTTGTCAC-3’; 3’-CTCTGGGTTGGCACACACTT-5’. IFNB: 5’-ATGACCAACAAGTGTCTCCTCC-3’; STING: 5’-

CCTGAGTCTCAGAACAACTGCC-3'; 3'- GGTCTTCAAGCTGCCCACAGTA-5'; 3'- GGAATCCAAGCAAGTTGTAGCTC-5'. PD-L1: 5'-TGCCGACTACAAGCGAATTACTG-3'; 3'- CTGCTTGTCCAGATGACTTCGG-5'. Mouse QRT-PCR primer sequences were: GAPDH: 5'-CATCACTGCCACCCAGAAGACTG-3'; 3'-ATGCCAGTGAGCTTCCCGTTCAG-5'. IFIT1: 5'-AGAGTCAAGGCAGGTTTCTG-3'; 3'-TGTGAAGTGACATCTCAGCTG-5'. IFI44: 5'-ATGCACTCTTCTGAGCTGGTGG-3'; 3'-TCAGATCCAGGCTATCCACGTG-5'. CCL5: 5'-CCTGCTGCTTTGCCTACCTCTC-3'; 3'-ACACACTTGGCGGTTCCTTCGA-5'. IFNB: 5'-AGCTCCAAGAAAGGACGAACAT-3'; 3'-GCCCTGTAGGTGAGGTTGATCT-5'. STING: 5'-GGTCACCGCTCCAAATATGTAG-3'; 3'-CAGTAGTCCAAGTTCGTGCGA-5'. RNA expression levels were normalized to GAPDH and calculated as fold change compared to control ($2^{-\Delta\Delta CT}$).

Detection of DNA in cytosolic extracts

2×10^6 cells were divided into two equal aliquots. One aliquot was resuspended in 500 µL of 50 µM NaOH and boiled for 30 minutes to solubilize DNA. 50 µL 1M Tris- HCl (pH 8.0) was added to neutralize the pH, and these extracts served normalization controls for total mtDNA. The second equal aliquots were resuspended in 500 µL buffer containing 150 mM NaCl, 50 mM HEPES (pH 7.4), and 20 µg/mL digitonin (D141; Sigma). The homogenates were incubated end over end for 10 minutes to allow selective plasma membrane permeabilization. Then centrifuged at 16,000 × g for 25 minutes at 4 °C and the cytosolic supernatants were harvested to remove the remaining cellular debris. DNA from whole cell lysates or cytosolic extract was quantified by QRT-PCR with gDNA primers and mtDNA primers. gDNA primers for QRT-PCR were: RPL13: 5'-GTAACCCGTTGAACCCCATT-3'; 3'-CCATCCAATCGGTAGTAGCG-5'; RNA18S: 5'-GTAACCCGTTGAACCCCATT-3'; 3'-CCATCCAATCGGTAGTAGCG-5'. mtDNA primers for QRT-PCR were: MT-ND1: 5'-CTCTTCGTCTGATCCGTCCT-3'; 3'-TGAGGTTGCGGTCTGTTAGT-5'; MT-ND2: 5'-GTAGACAGTCCCACCCTCAC-3'; 3'- TTGATCCCGTTTCGTGCAAG-5. gDNA/mtDNA CT values of the cytosolic fractions were normalized to gDNA abundance for whole-cell extracts to account for the variations of cell number among samples.

Western blot

Protein was extracted from cells or tissues using RIPA lysis buffer (Beyotime Biotechnology) in the presence of protease (Sigma; 11697498001) and phosphatase inhibitors (ThermoFisher; A32957). Protein concentration was evaluated using BCA (ThermoFisher; 23225). 10-20 µg proteins were loaded onto 10% SDS/PAGE gel and run. After electrophoresis, proteins were transferred into 0.45 µm PVDF membrane (GE Healthcare; 10600023). After transfer, the membranes were blocked using 5%

milk for 1 hour at room temperature. After blocking, the membranes were blotted with specific primary antibodies, including GAPDH (1:5000; Proteintech; 1E6D9), CGAS (1:1000; Cell Signaling Technology; 15102S), STING (1:1000; Cell Signaling Technology; 13647S), Phospho-STING (Ser366) (1:1000; Cell Signaling Technology; 50907T), IRF3 (1:1000; Cell Signaling Technology; 11904S), Phospho-IRF3 (Ser396) (1:1000; Cell Signaling Technology; 29047S) and PD-L1 (1:1000; Proteintech; 17952-1-AP) at 4 ℃ overnight. The membranes were then incubated with appropriate HRP-conjugated goat anti-rabbit secondary antibody (1:10000; BIOTECH WELL; WB3177) or HRP-conjugated goat anti-mouse secondary antibody (1:10000; BIOTECH WELL; WB0176) at 37 ℃ for 1 hour, and were visualized by Immobilon Western Chemiluminescent HRP Substrate (Millipore; WBKLS0500). Images of protein bands were taken by Tanon 5200 system. For quantification of the western blot results, densitometry intensity of western blot bands was analyzed using ImageJ software.

Flow cytometry

LNCaP and PC3 cells were firstly treated with bicalutamide, docetaxel, or bicalutamide plus docetaxel for 24 hours. Then, cells were fixed and permeabilized with eBiosience™ Foxp3/Transcription Factor Staining Buffer Set (Thermo Fisher; 00-5523-00) according to the provided manufacturer’s instructions. After fix and permeabilization, cells were incubated with 10% goat serum (Beyotime; C0265) for 15 minutes at room temperature. After blocking, the cells were incubated with the following primary antibodies for 30 minutes at room temperature: Phospho-STING (Ser366) (1:200; Cell Signaling Technology; 50907T), Phospho-IRF3 (Ser396) (1:200; Cell Signaling Technology; 29047S). Finally, cells were incubated with Goat anti-Rabbit IgG Alexa Fluor 488 (1:1000; Thermo Fisher; A-11008) for 30 minutes at room temperature. Data were acquired on a LSRFortessa flow cytometer (BD Biosciences) and further analyzed with FlowJo software.

Plasmid constructs

Human STING single-guide RNA (sgRNAs) and mouse STING short-hairpin RNA (shRNA) were cloned into lentiCRISPR-V2 and PLKO.1 plasmid, respectively. All plasmids were verified by DNA sanger sequencing. To prepare lentiviral particles, HEK-293T cells were transfected with plasmids using Hieff Trans™ Liposomal Transfection Reagent (YEASEN, 40802ES02). Prostate cancer cells were infected with lentiviral particles expressing either lentiCRISPR-V2-STING-KO or PLKO.1-shSTING for 72 hours and then selected using Puromycin at a concentration of 2 mg/mL. The sequences used were 5’-AGAGCACACTCTCCGGTACC-3’ for lentiCRISPR- V2-STING-KO and 5’-ATGATTCTACTATCGTCTTAT-3’ for PLKO.1-shSTING#1 and 5’-CAACATTCGATTCCGAGATAT-3’ for PLKO.1-shSTING#2.

In vitro cell growth assay

RM1 scramble (2000 cells/well) and RM1 shSTING (2000 cells/well) cells were seeded onto a 96-well plate and cultured for 24, 48, and 60 hours. The cell number at each time point was determined by CCK8 assay (Vazyme; A311-02).

Table S1. Characteristics of prostate cancer patients who were treat naive (n = 41) or received chemohormonal therapy (n = 45).

		n (%)		P-value
Characteristic	Category	OP (n = 41)	NCHT (n = 45)	/
Age	Median	69 years	65 years	0.0011
PSA level	Median	59.66 ng/mL	94.21 ng/mL	0.00060
Tumor status	T1	0 (0.00)	0 (0.00)	1.00
	T2	6 (14.63)	14 (31.11)	0.071
	T3	35 (85.37)	18 (40.00)	<0.0001
	T4	0 (0.00)	13 (28.89)	0.00010
Nodal status	N0	31 (75.61)	26 (57.78)	0.081
	N1	10 (24.39)	19 (42.22)	0.081
Gleason score	6	2 (4.88)	1 (2.22)	0.93
	7	14 (34.15)	16 (35.56)	0.89
	8	14 (34.15)	12 (26.67)	0.45
	9	11 (26.83)	15 (33.33)	0.51
	10	0 (0.00)	1 (2.22)	1.00
Histology	Adenocarcinoma	41 (100.00)	45 (100.00)	/
Neoadjuvant	ADT&Docetaxel	0 (0.00)	45 (100.00)	/

ADT: Androgen-deprivation therapy; PSA: Prostate-specific antigen

Table S2. Changes in clinicopathological characteristics of 11 prostate cancer patients before and after chemohormonal therapy.

Patient ID	Age (Years)	Pre-PSA (ng/mL)	Post-PSA (ng/mL)	Pre-T stage	Post-T stage	Pre-N stage	Post-N stage
P_1	66	92.79	4.57	3b	0	0	0
P_2	61	4.08	0.01	3b	2c	0	0
P_3	77	38.86	0.06	4	0	0	0
P_4	64	151	0.05	3b	2c	0	0
P_5	63	94.21	0.17	3b	2c	0	0
P_6	68	235	0.27	3b	3b	0	0
P_8	74	182	0.87	3b	3b	0	0
P_9	61	38	0.09	3a	2c	1	1
P_12	55	197	0.43	3b	2c	1	1
P_14	69	227.87	1.42	3b	3b	1	0
P_16	62	100	0.05	3a	2c	0	0

PSA: Prostate-specific antigen

Table S3. Signatures related gene sets used in analysis.

Antigen presentation		Batf3-dendritic cell signature	CYT score	CD8+ effector T cell signature	T cell inflamed signature
HLA-DOA	IFNA16	BATF3	GZMA	CD8A	CXCR6
HLA-DOB	KIR2DL5A	IRF8	PRF1	CD8B	TIGIT
KLRC3	HSPA6	THBD		IFNG	CD27
KLRD1	HLA-DRB4	CLEC9A		PRF1	CD274
KLRC1	CD4			PDCD1LG2	
KLRC2	KIR2DL1			LAG3	
RFXAP	HLA-DRB5			NKG7	
RFX5	LTA			PSMB10	
IFNA5	KIR3DL3			CMKLR1	
IFNA4	HLA-DRB3			IDO1	
IFNA2	TAP2			CCL5	
IFNA1	KIR2DS4			CXCL9	
LGMN	TAPBP			HLA-DQA1	
PSME3	CD8A			CD276	
CTSS	CD8B			HLA-DRB1	
HLA-C	TAP1			STAT1	
HLA-B	KIR2DS3			HLA-E	
HLA-DMB	HSPA8				
HLA-DMA	KIR2DL4				
HLA-A	CANX				
HSPA1L	KIR2DS1				
HSPA1B	KIR2DL2				
HSPA2	KIR2DL3				
KIR2DS5	KLRC4				
HLA-G	NFYC				
KIR3DL1	HSP90AA1				
KIR3DL2	NFYA				
HSPA1A	NFYB				
RFXANK	HLA-F				
CREB1	CTSB				
IFNA17	HLA-E				
HSPA5	CALR				
CD74	HLA-DPA1				
HSPA4	HLA-DPB1				
IFNA21	HLA-DQA1				
CTSL	PDIA3				
IFNA6	HLA-DQA2				
IFI30	HLA-DQB1				
IFNA7	PSME1				
IFNA8	PSME2				
IFNA10	HSP90AB1				
IFNA13	B2M				
CIITA	HLA-DRB1				
IFNA14	HLA-DRA				

Fig. S1

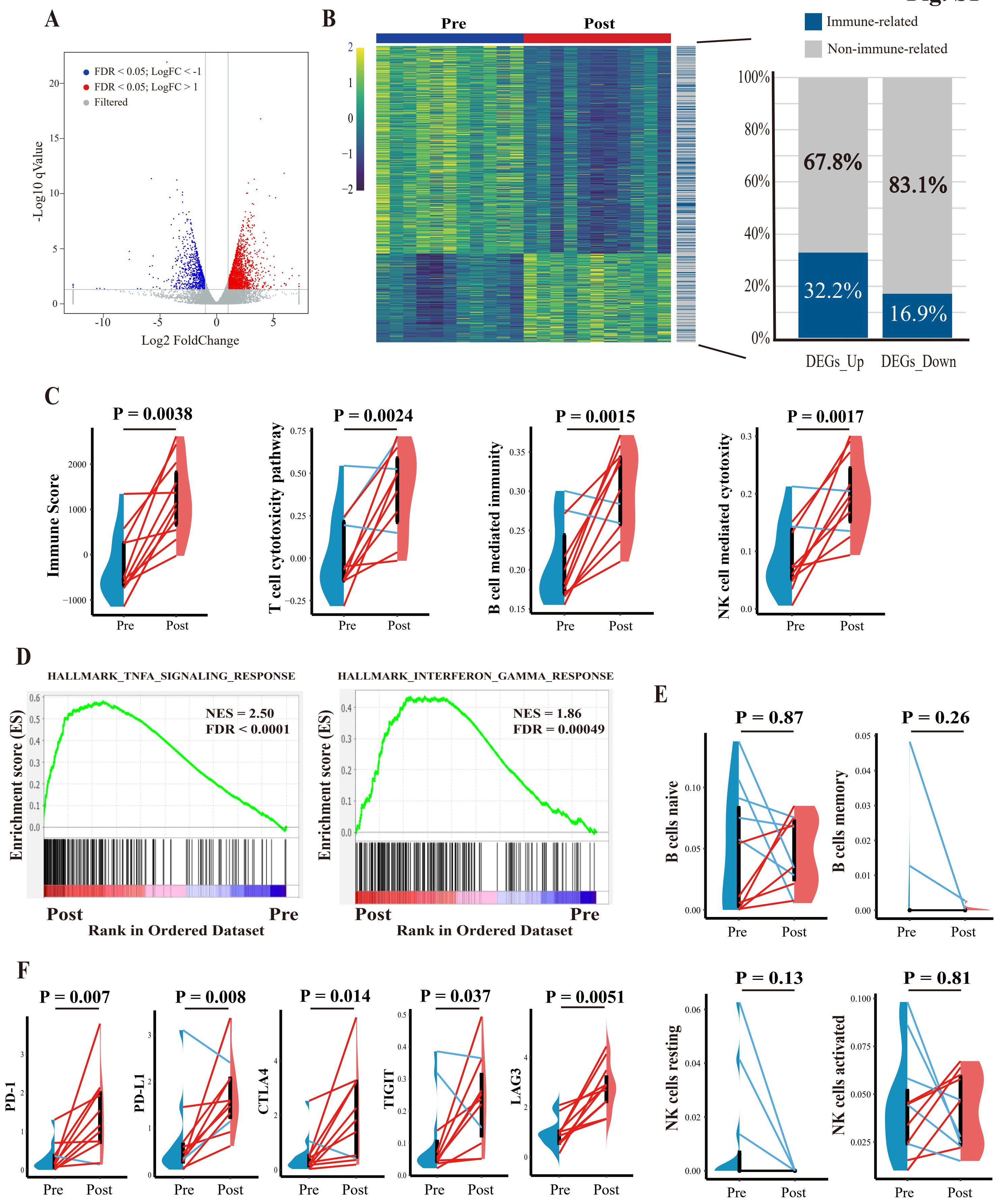

Fig. S1. Transcriptome and gene expression profiling of tumors before and after chemohormonal therapy in prostate cancer patients. **A.** Volcano plot displays differentially expressed genes between paired before and after chemohormonal therapy tumor samples. **B.** Heatmap showing the differential expressed genes between paired before and after chemohormonal therapy tumor samples. Right panel highlights the proportion of immune-related differential expressed genes in all differential expressed genes. **C.** Changes in Immune Score, T cell cytotoxicity pathway, B cell-mediated immunity, and NK cell-mediated cytotoxicity between paired before and after chemohormonal therapy tumor samples. **D.** Gene set enrichment analysis indicates post-chemohormonal therapy tumor samples are characterized of TNFA signaling response and interferon gamma response compared with pretreatment samples. **E.** Changes in fractions of naive B cells, memory B cells, resting NK cells, and activated NK cells between paired before and after chemohormonal therapy tumor samples. **F.** Changes in expression levels of PD-1, PD-L1, CTLA4, TIGIT, and LAG3 between paired before and after chemohormonal therapy tumor samples. Each point represents an independent sample. Data were presented as mean values ± SEM. Paired data were analyzed using the paired t-test or Wilcoxon paired rank test.

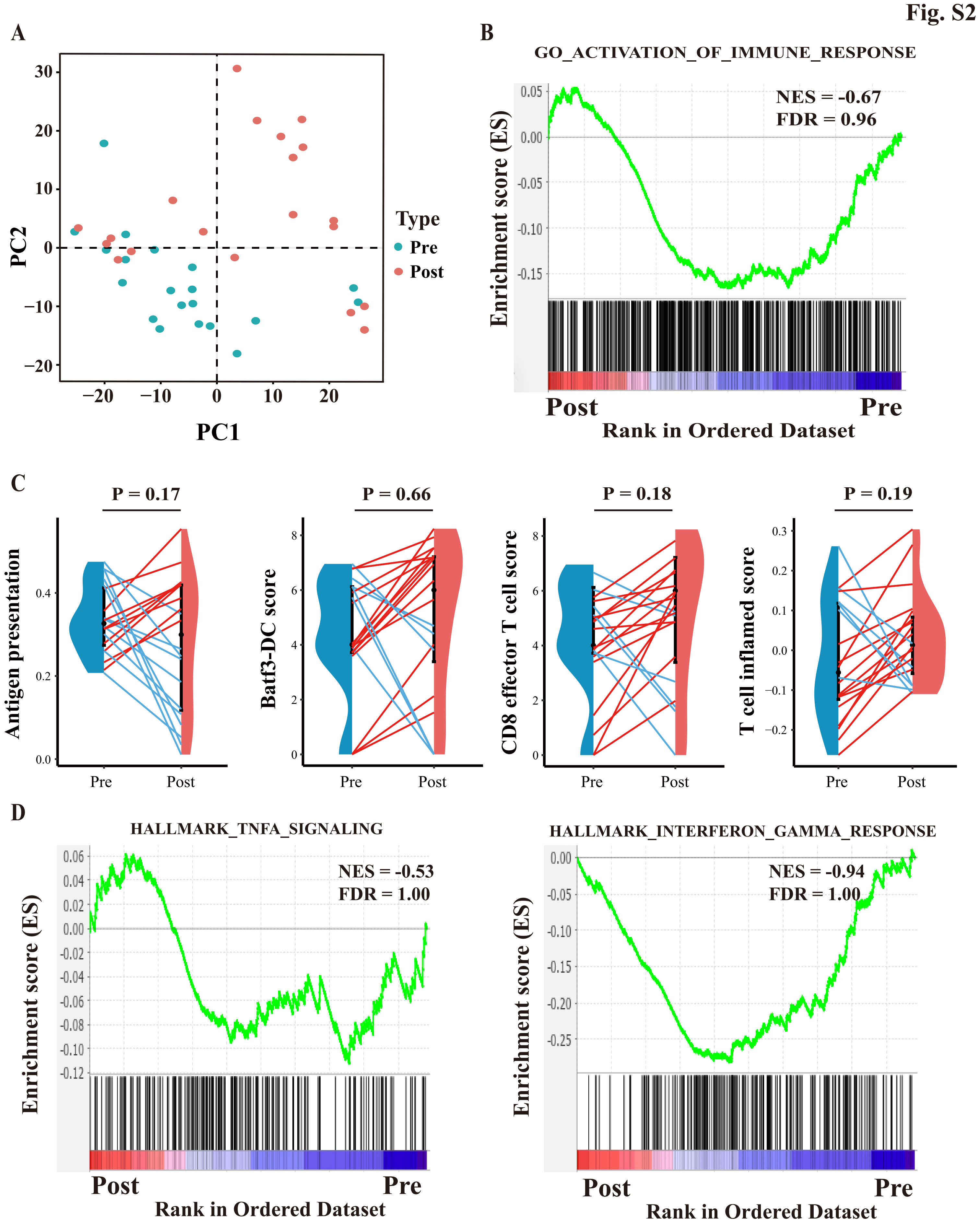

Fig. S2. Gene signatures of tumors before and after hormonal therapy in prostate cancer patients. **A.** Principal component analysis of transcriptomic data from paired before and after hormonal therapy tumor samples. Each dot represents a patient sample that is colored on the basis of treatment (blue, pretreatment; red, posttreatment). **B.** Gene set enrichment analysis for gene sets of immune response between paired before and after hormonal therapy tumor samples. **C.** Changes in antigen presentation score, Batf3-DC score, CD8+ effector T cell score, and T cell inflamed signature score between paired before and after hormonal therapy tumor samples. **D.** Gene set enrichment analysis for gene sets of TNFα signaling response and interferon gamma response between paired before and after hormonal therapy tumor samples. Each point represents an independent sample. Data were presented as mean values ± SEM. Paired data were analyzed using the paired t-test or Wilcoxon paired rank test.

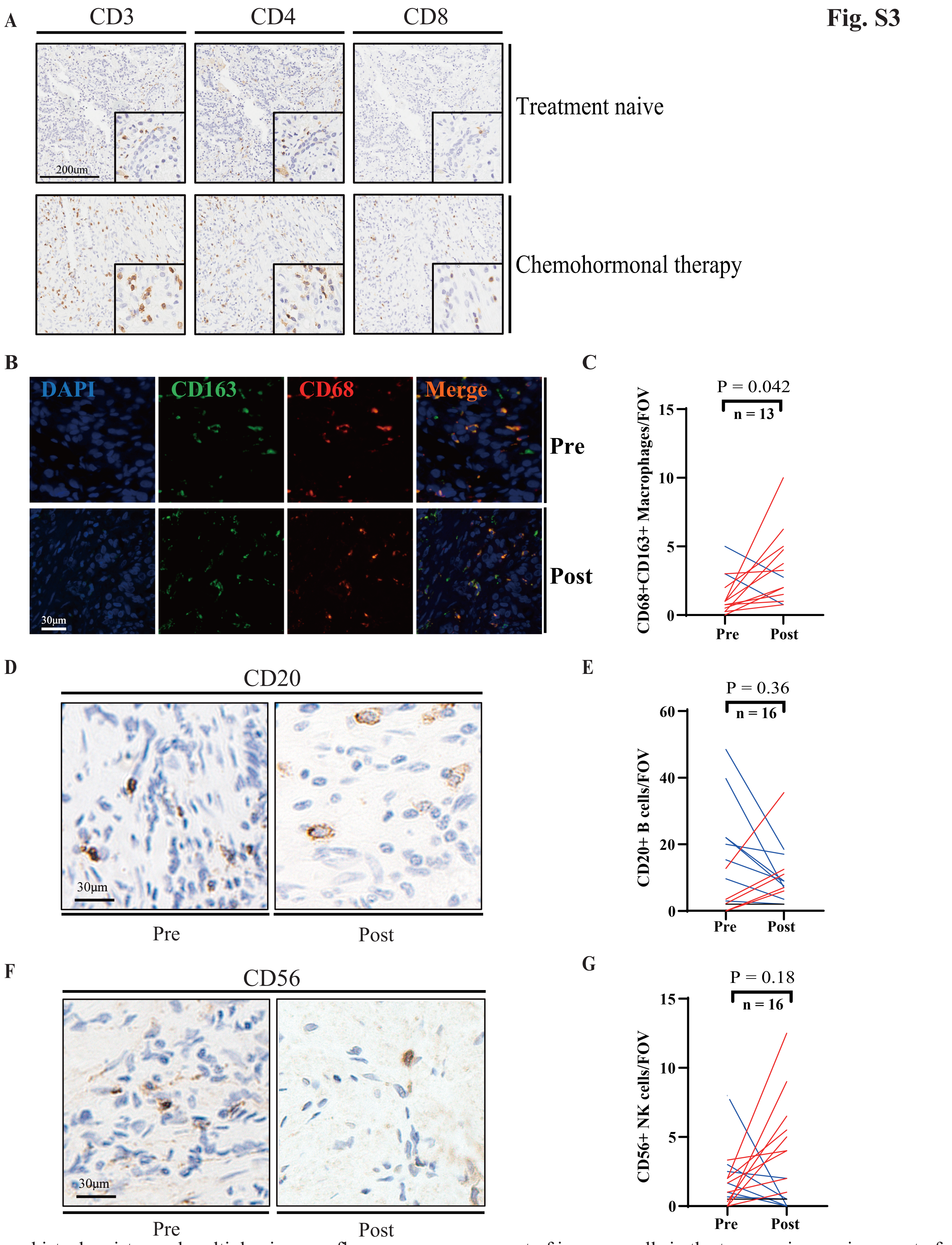

Fig. S3. Immunohistochemistry and multiplex immunofluorescence assessment of immune cells in the tumor microenvironment of pre- and post-chemohormonal therapy tumor samples. **A.** Representative immunohistochemistry staining images of $CD3^{+}$ T cells, $CD4^{+}$ T cells, and $CD8^{+}$ T cells between treatment naive and chemohormonal therapy tumor samples. **B.** Representative fluorescence images of immunolabeled macrophages ($CD163^{+}CD68^{+}$) from paired before and after chemohormonal therapy tumor samples. **C.** Changes in the densities of immunolabeled macrophages ($CD163^{+}CD68^{+}$) in (B) between paired before and after chemohormonal therapy tumor samples. FOV, field of view. **D.** Representative immunohistochemistry staining images of $CD20^{+}$ B cells from paired before and after chemohormonal therapy tumor samples. **E.** Changes in the densities of $CD20^{+}$ B cells in (D) between paired before and after chemohormonal therapy tumor samples. FOV, field of view. **F.** Representative immunohistochemistry staining images of $CD56^{+}$ NK cells from paired before and after chemohormonal therapy tumor samples. **G.** Changes in the densities of $CD56^{+}$ NK cells in (F) between paired before and after chemohormonal therapy tumor samples. FOV, field of view. Each point represents an independent sample. Paired data were analyzed using the paired t-test or Wilcoxon paired rank test.

Fig. S4

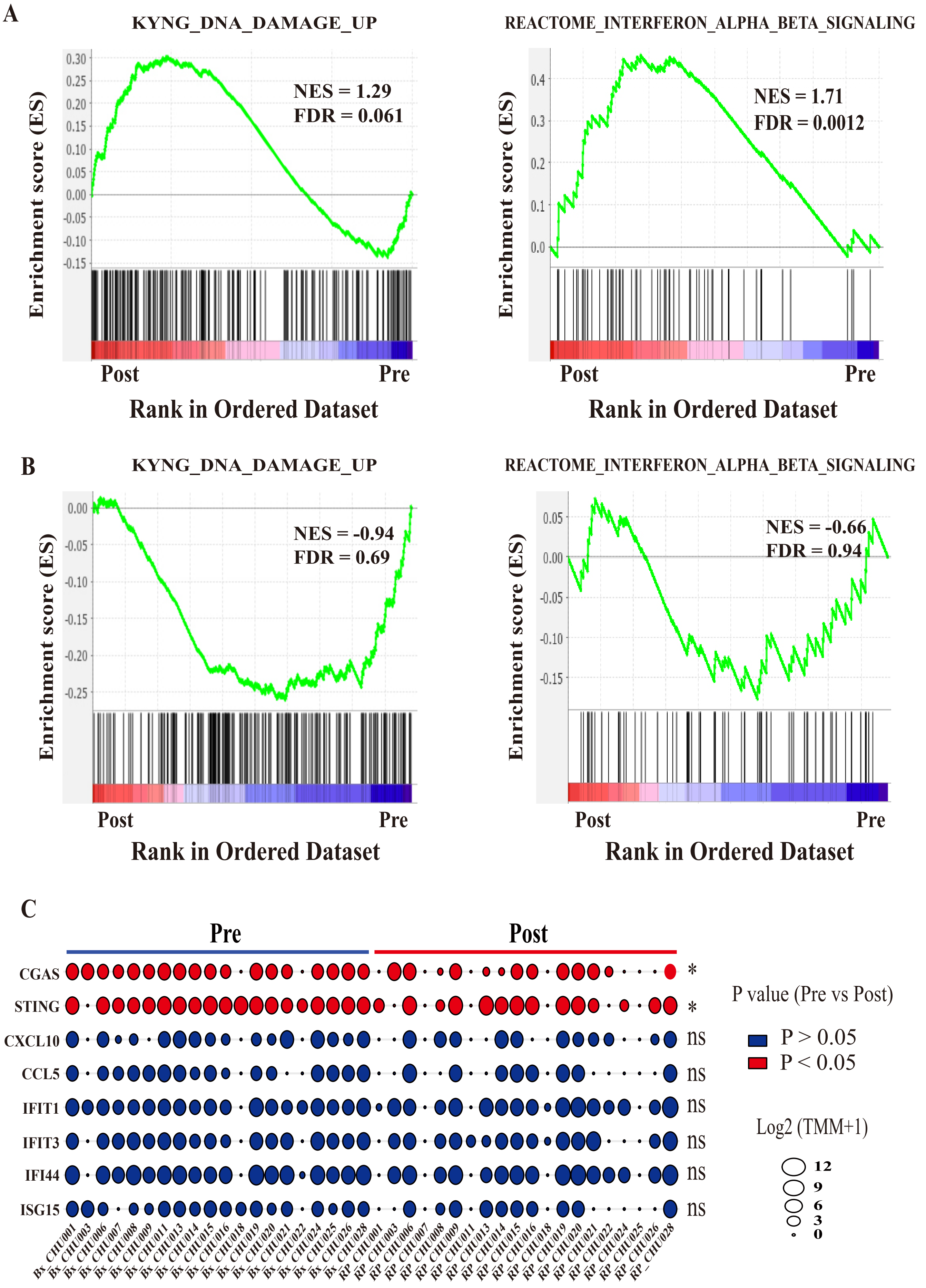

Fig. S4. Chemohormonal therapy activates the cGAS/STING pathway in prostate cancer. **A.** Gene set enrichment analysis for gene sets of DNA damage and interferon signaling between paired before and after chemohormonal therapy tumor samples. **B.** Gene set enrichment analysis for gene sets of DNA damage and interferon signaling between paired before and after hormonal therapy tumor samples. **C.** Bubble plot showing the transcriptome analysis of the cGAS/STING pathway-related genes for paired pre- and post-hormonal therapy tumor samples. Bubble size reflects the relative expression level of gene. Bubble color reflects the P value. Each point represents an independent sample. Paired data were analyzed using the paired t-test or Wilcoxon paired rank test.

Fig. S5

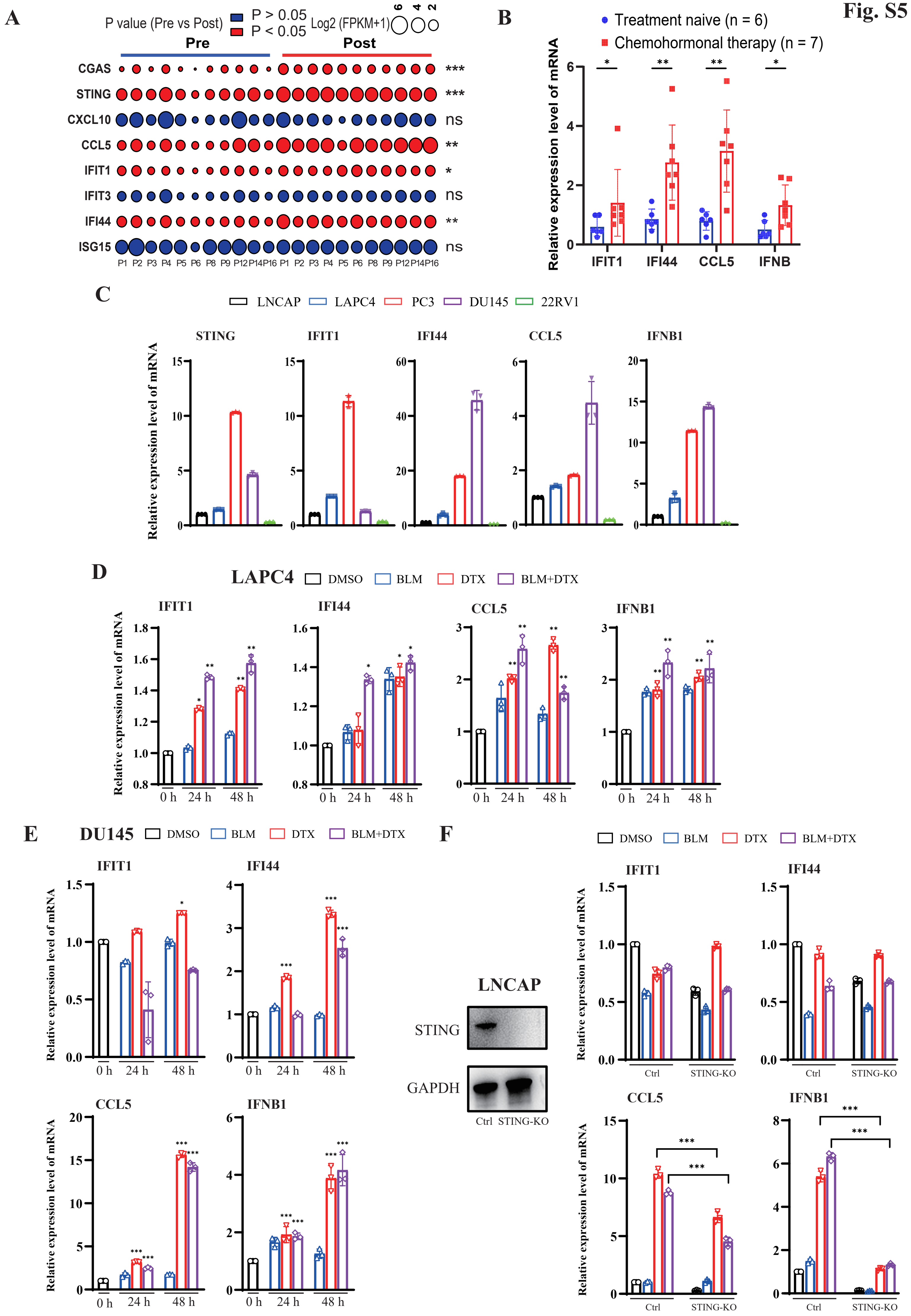
A
P value (Pre vs Post)
P > 0.05
P < 0.05
Log2 (FPKM+1)
6 4 2
Pre
Post
CGAS ***
STING ***
CXCL10 ns
CCL5 **
IFIT1 *
IFIT3 ns
IFI44 **
ISG15 ns
P1 P2 P3 P4 P5 P6 P8 P9 P12 P14 P16 P1 P2 P3 P4 P5 P6 P8 P9 P12 P14 P16
B
Treatment naive (n = 6)
Chemohormonal therapy (n = 7)
Relative expression level of mRNA
IFIT1
IFI44
CCL5
IFNB
C
LNCAP
LAPC4
PC3
DU145
22RV1
STING
IFIT1
IFI44
CCL5
IFNB1
Relative expression level of mRNA
D
LAPC4
DMSO
BLM
DTX
BLM+DTX
IFIT1
IFI44
CCL5
IFNB1
Relative expression level of mRNA
0 h
24 h
48 h
E
DU145
DMSO
BLM
DTX
BLM+DTX
IFIT1
IFI44
CCL5
IFNB1
Relative expression level of mRNA
0 h
24 h
48 h
F
LNCAP
STING
GAPDH
Ctrl STING-KO
DMSO
BLM
DTX
BLM+DTX
IFIT1
IFI44
CCL5
IFNB1
Relative expression level of mRNA
Ctrl
STING-KO

Fig. S5. Combination of docetaxel and androgen-deprived treatment activates the cGAS/STING pathway in prostate cancer cells. **A.** Bubble plot showing the transcriptome analysis of the cGAS/STING pathway-related genes for paired pre- and post-chemohormonal therapy tumor samples. Bubble size reflects the relative expression level of gene. Bubble color reflects the P value. **B.** Differences of cGAS/STING pathway downstream immune genes between treatment naive and chemohormonal therapy tumor samples. **C.** QPCR analysis of the cGAS/STING pathway-related genes (STING, IFIT1, IFI44, CCL5, and IFNB1) in different prostate cancer cell lines. **D-E.** QPCR analysis of cGAS/STING pathway downstream immune genes in LAPC4 cells (D) and DU145 cells (E) after treatment with DMSO, bicalutamide (BLM), docetaxel (DTX), or bicalutamide plus docetaxel (BLM+DTX) for the indicated time. **F.** QPCR analysis of cGAS/STING pathway downstream immune genes in LNCaP cells with or without STING knockout after treatment with DMSO, bicalutamide (BLM), docetaxel (DTX), or bicalutamide plus docetaxel (BLM+DTX) for 48 h. Western blot showing the knockout efficiency of STING in LNCaP cells. Data were presented as mean values ± SEM. Unpaired data were analyzed using the t-test or Wilcox rank sum test. *, $P < 0.05$; **, $P < 0.01$; ***, $P < 0.001$.

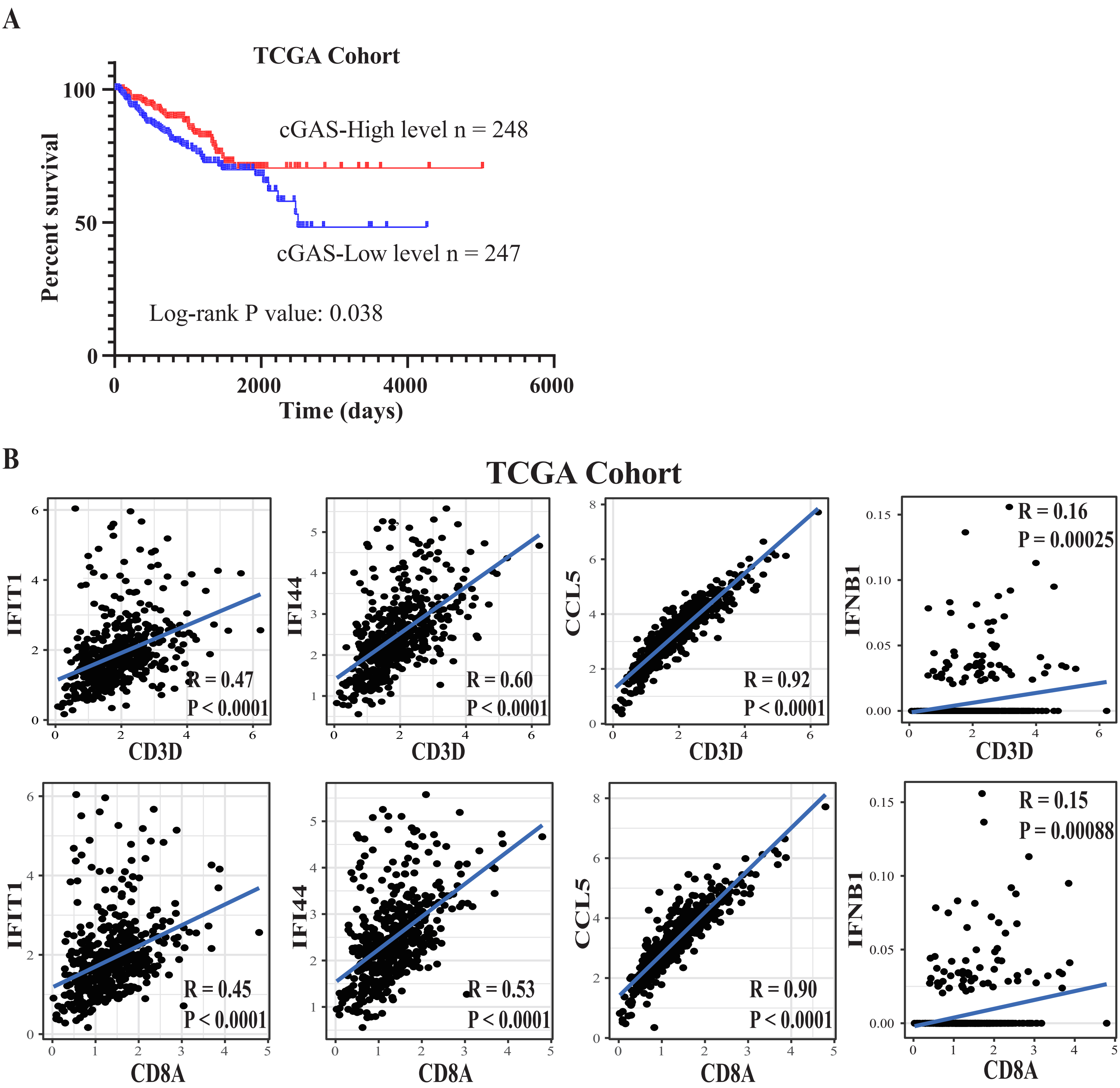

Fig. S6. Analysis of the cGAS/STING pathway in prostate cancer cohort. **A.** Kaplan–Meier survival analysis of cGAS in the TCGA prostate cancer patient cohort. **B.** Expression correlation of CD3D and CD8A mRNA with cGAS/STING pathway downstream immune genes (IFIT1, IFI44, CCL5, and IFNB1) in the TCGA prostate cancer patient cohort.

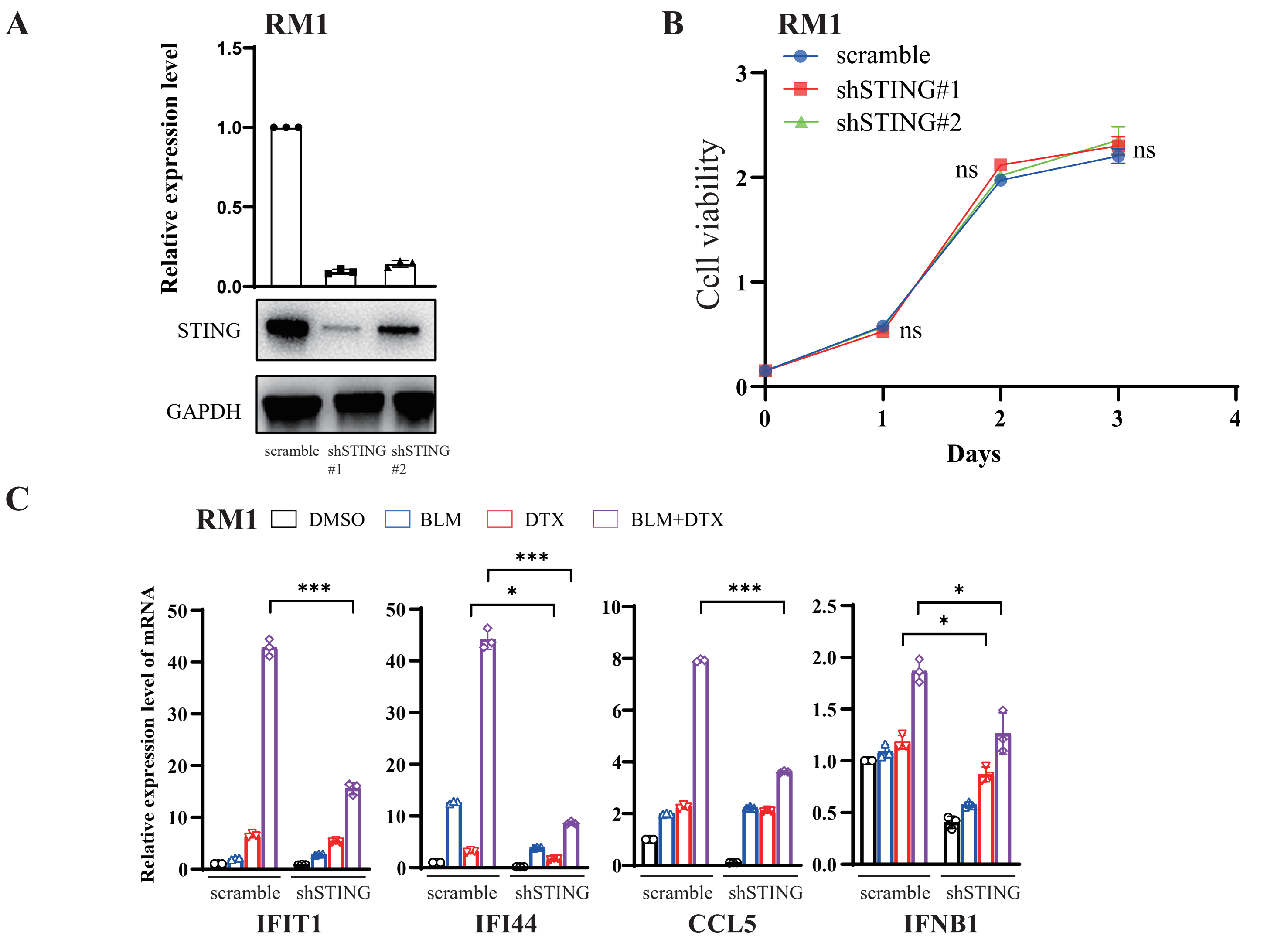

Fig. S7. Chemohormonal therapy sensitizes prostate cancer-bearing mice to PD1-bloackade therapy. **A.** QPCR and Western blot showing the knockdown efficiency of STING in RM1 cells. **B.** Cell viability analysis for RM1 cells (scramble or shSTING). **C.** QPCR analysis of cGAS/STING downstream immune genes in RM1 cells (scramble or shSTING) after treatment with DMSO, bicalutamide (BLM), docetaxel (DTX), or bicalutamide plus docetaxel (BLM +DTX) for 48 h. Each point represents an independent experiment. Data were presented as mean values ± SEM. Unpaired data were analyzed using the t-test or Wilcox rank sum test. *, P < 0.05; **, P < 0.01; ***, P < 0.001.

Fig. S8

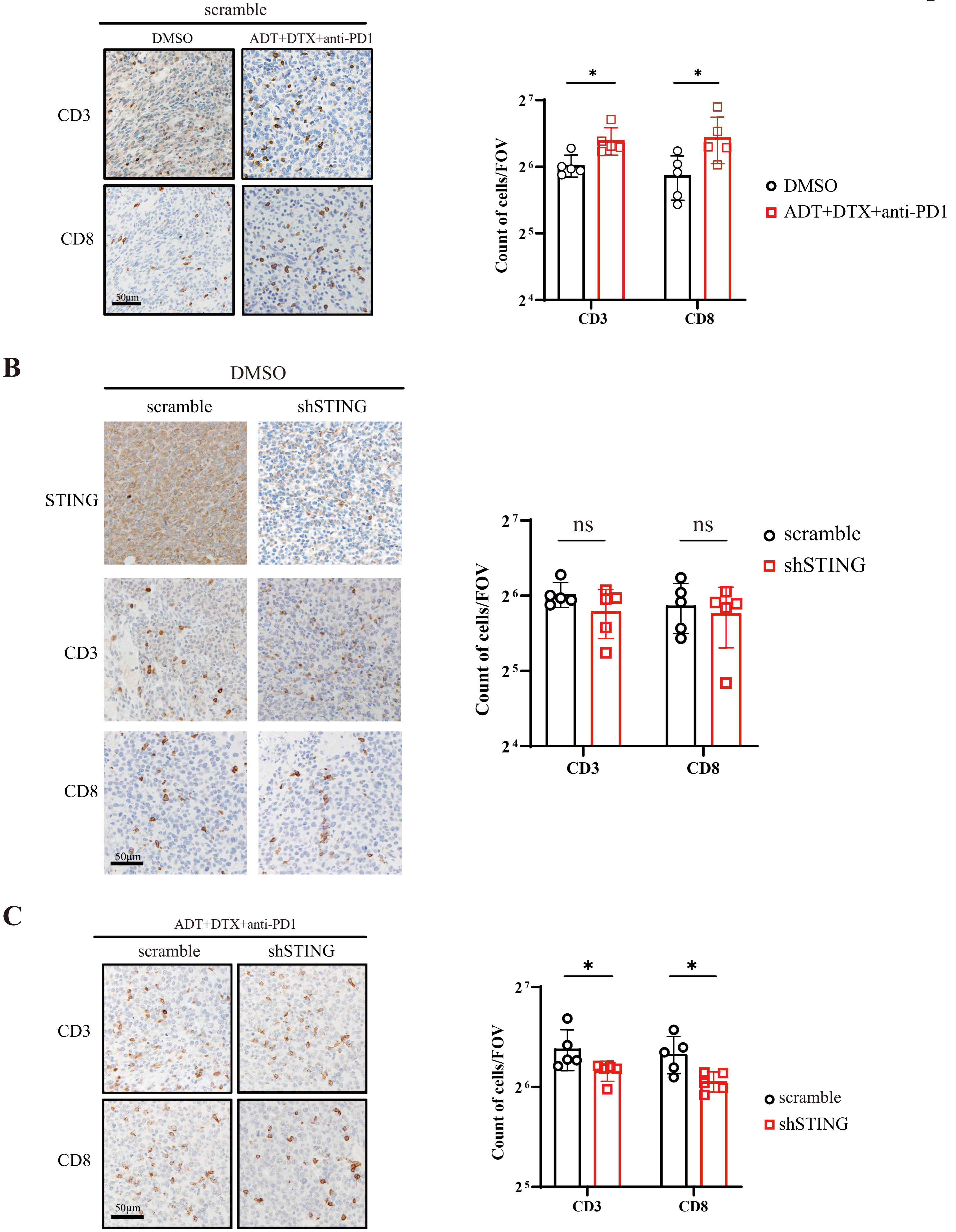

Fig. S8. Chemohormonal therapy activates immune response to suppress xenografted tumors growth. **A**. Representative immunohistochemical staining images and quantification of the percentage of CD3 and CD8 positive cells in RM1 (scramble) tumor xenografts after bicalutamide+docetaxel treatment. **B.** Representative immunohistochemical staining images and quantification of the percentage of CD3 and CD8 positive cells in RM1 (scramble and shSTING) tumor samples after DMSO treatment. **C.** Representative immunohistochemical staining images and quantification of the percentage of CD3 and CD8 positive cells in RM1 (scramble and shSTING) tumor xenografts after anti-PD-1+ bicalutamide+docetaxel treatment. Each point represents an independent sample. Data were presented as mean values ± SEM. Unpaired data were analyzed using the t-test or Wilcox rank sum test. *, $P < 0.05$; **, $P < 0.01$; ***, $P < 0.001$.
